# Complex genetic dependencies among growth and neurological phenotypes in healthy children: Towards deciphering developmental mechanisms

**DOI:** 10.1371/journal.pone.0242684

**Published:** 2020-12-03

**Authors:** Lisa Uechi, Mahjoubeh Jalali, Jayson D. Wilbur, Jonathan L. French, N. L. Jumbe, Michael J. Meaney, Peter D. Gluckman, Neerja Karnani, Nikita A. Sakhanenko, David J. Galas, on behalf of the GUSTO study group

**Affiliations:** 1 Pacific Northwest Research Institute, Seattle, Washington, United States of America; 2 Metrum Research Group, Tariffville, CT, United States of America; 3 Pharmactuarials LLC, Mountain View, CA, United States of America; 4 Douglas Mental Health University Institute, McGill University, Montreal, QC, Canada; 5 Child and Brain Development Program, Canadian Institute for Advanced Research (CIFAR) Institute, Toronto, Canada; 6 Centre for Human Evolution, Adaptation and Disease, Liggins Institute, University of Auckland, Auckland, New Zealand; 7 Department of Biochemistry, Yong Loo Lin School of Medicine, National University of Singapore, Singapore, Singapore; 8 Brenner Centre for Molecular Medicine, National University of Singapore, Singapore, Singapore; University of Iceland, ICELAND

## Abstract

The genetic mechanisms of childhood development in its many facets remain largely undeciphered. In the population of healthy infants studied in the Growing Up in Singapore Towards Healthy Outcomes (GUSTO) program, we have identified a range of dependencies among the observed phenotypes of fetal and early childhood growth, neurological development, and a number of genetic variants. We have quantified these dependencies using our information theory-based methods. The genetic variants show dependencies with single phenotypes as well as pleiotropic effects on more than one phenotype and thereby point to a large number of brain-specific and brain-expressed gene candidates. These dependencies provide a basis for connecting a range of variants with a spectrum of phenotypes (pleiotropy) as well as with each other. A broad survey of known regulatory expression characteristics, and other function-related information from the literature for these sets of candidate genes allowed us to assemble an integrated body of evidence, including a partial regulatory network, that points towards the biological basis of these general dependencies. Notable among the implicated loci are RAB11FIP4 (next to NF1), MTMR7 and PLD5, all highly expressed in the brain; DNMT1 (DNA methyl transferase), highly expressed in the placenta; and PPP1R12B and DMD (dystrophin), known to be important growth and development genes. While we cannot specify and decipher the mechanisms responsible for the phenotypes in this study, a number of connections for further investigation of fetal and early childhood growth and neurological development are indicated. These results and this approach open the door to new explorations of early human development.

## 1. Introduction

An estimated 165 million children under 5 years of age sustain stunted growth, and more than 200 million children suffer impaired neurocognitive development in developing countries around the world [1, 2, 76]. These effects, caused by a variety of factors, lead to their subsequent loss of health, cognitive skills and productivity. Although many of these factors are potentially preventable, to deal them we need a deeper understanding of the mechanisms governing child development. We have focused here on elucidating some of the key biological determinants of growth and neurocognitive development in healthy children, and their interactions. As part of the program for the Healthy Birth, Growth, and Development (HBGDKi) effort of the Bill and Melinda Gates Foundation, we have examined neurological development, fetal and early childhood growth data, and the genotypes of infants in the Growing Up in Singapore Towards healthy Outcomes (GUSTO) study [3, 4]. By analyzing detailed phenotypic, longitudinal developmental data and genetic data on this population of normal, healthy children using our information theory-based methods, we have detected multiple complex dependencies among these variables.

While it is common to look for genetic variants that affect single phenotypes (pairwise genetic effects—one locus, one phenotype, as is done for disease or pathology analysis) the richness of information in pleiotropic effects (one locus, two or more phenotypes) has been largely neglected and provides additional information.

Pleiotropy is well-known in humans, with clear effects reported by [5–9] for example, and is, in fact, rather common. Every known genetic "syndrome" that has been identified with variants in specific genes (see the OMIM database for very long lists of these "syndromes") represents a pleiotropic effect. This is true because a "syndrome" is defined as a collection of phenotypes that share influences from variants in a particular gene. Thus, the genetic cause of a specific syndrome is by its definition a pleiotropic effect. Realizing that a single genetic variant affecting more than one phenotype implies a commonality in the effect on more than one mechanistic pathway, those pathways affecting the respective phenotypes, implies that such genetic variants carry a deeper kind of information than the usual single locus- single phenotype dependencies. This sharing of genetic influences indicates a connection between the mechanistic pathways, which is a strength of our approach.

In the work reported here, we have analyzed both single locus-single phenotype and pleiotropic effects.

Pleiotropy, the phenomenon where a genetic region or locus confers risk to more than one trait1, is widely observed for many diseases and traits [2], especially cancers [3], autoimmune [4] and psychiatric [10, 11] disorders. It has also been observed in seemingly unrelated traits; for instance, early-onset androgenetic alopecia and Parkinson's disease, Crohn's disease and Parkinson's disease [13], and coronary artery disease and tonsillectomy [5–9].

While our methods can analyze any number of interacting variables, we are limited by sample numbers. We have also looked for possible interactions between the genetic loci identified. These dependencies point to various biological pathways contributing to growth and neurocognitive development.

In an effort to identify factors contributing to the effects on growth and neurocognitive development, we reasoned that multiple layers of analysis, beginning with evidence for dependency among phenotypic and genetic variables, followed by a knowledge-based approach from previous work in the literature, such as known associations of genetic loci with expression in certain tissues, commonalities in regulatory pathways among associated genes, and other functional information, could potentially uncover subtle effects that conventional methods might not detect [10–12]. This effort was enabled by our analysis methods that can reliably detect three-variable dependency [13–15], described in detail in the Methods section 4.3. Application of our three-variable dependency method did indeed allow identification of a number of candidate genes that exhibit no significant pairwise dependence with a single phenotype, and would therefore be missed altogether by common genetic association methods. These can be characterized as fundamentally pleiotropic loci.

The specific purposes of this overall effort were rather different from most genetic studies. Rather than searching for a handful of highly significant causal genes (which is typical for a disease research) we focused on attempting to reveal biological determinants of growth and neurocognitive development in healthy children by finding multiple less significant genetic correlates, and to elucidate the specific dependencies among neurological development, physical development and SNPs of infants in the GUSTO study. We wished to identify candidate genomic regions, genes and/or regulatory interactors that may be involved in these developmental processes. Since synergy of biological effects is common, we sought to identify as many genetic signals as possible, including some that exhibit relatively low significance by themselves, in order to collect multiple pieces of evidence that might collectively point to a set of candidate genes or loci within the genome, and then to biological pathways or networks. The compilation of extensive regulatory and gene expression data on implicated genes allowed us to implicate a number of developmental processes. Notable were the large number of connections to brain-specific and brain-related expression and processes known to affect brain phenotypes. While the wide range of information that is integrated in this analysis suggests several intriguing conclusions, the outstanding limitation of this study is that, to our knowledge, there is no comparable data set that can be used for cross validation. Nevertheless, the resulting candidate dependencies identified by our method are indirectly validated using multiple public databases.

In the Results section, we present the outcomes of a consecutive set of analyses. We examined dependencies between longitudinal growth parameters of head circumference, and neurological development scores of two-year olds; next, we looked at the genetic dependencies of each of these phenotypes separately; and finally, determined the pleiotropic three-way dependencies among phenotypes and specific SNP's. Significant dependencies were found in each of these steps, and these sets of genetic variants collectively implicated some of the same processes.

Nomenclature:

- Bayley phenotypes (or simply Bayley) = Bayley scale scores
- Adaptive = composite Adaptive Bayley scale score
- Social-emotional = composite Social-emotional Bayley scale score
- Motor = composite Motor Bayley scale score
- Cognitive = composite Cognitive Bayley scale score
- Language = composite Language Bayley scale score
- Growth phenotypes = growth model parameters linf, lambda, alpha
- Two-way (or pairwise) dependence measure = mutual information
- Three-way dependence measure = Delta3

## 2. Results

### 2.1. Relationships between fetal and early childhood growth and neurological development

To determine if there were effects on neurological development of fetal and early childhood growth profiles, we looked for dependencies among various data variables that represented aspects of these processes. We used our information theory methods, which assume no models (see Methods Section 4.3) [13–15], to examine dependencies between growth phenotypes and neurological measurements. Our initial attempts to detect dependencies between the raw growth measurement data points (head circumference) and neurological measurements (e.g., Bayley phenotypes) of infants at two years of age led to relatively poor statistics, probably caused by characteristics of the growth data including the variable times of growth measurement, the noise in these single point measurements and missing data, particularly in the fetal growth data sets. To resolve this problem, we fit the growth data of the entire population to a parameterized Gompertz-like model resulting in a population mean curve and we then estimated individual deviations and used the model parameters estimates for each subject as the growth phenotype variables to examine for dependency together with the neurological data (see Methods section 4.2.3 for details and fit statistics). The growth model involves three parameters that describe the final growth limit, a growth rate parameter and the non-linearity of growth deceleration. These three growth curve parameters were analyzed for two- and three-way dependencies with the neurological phenotypes, including Bayley [16], Infant Toddler Social Emotional Assessment (ITSEA) and Child Behavior Check List (CBCL).

The pairwise dependence measures and the three-way dependence measure (see Methods section 4.3) were calculated for 1073 subjects and permutation tests were performed to generate p-values (described in Methods section 4.4). We found a number of effects in both the two-variable and three-variable cases. As shown in Table 1, the strongest effect (lowest *p*-value) for two-way dependencies was clearly between the composite Cognitive Bayley phenotype and any growth parameter. The correlations indicated between phenotypes suggest that there is a relationship and, hence, the possibility of common causes. Furthermore, such correlations suggest that stringent corrections for multiple tests may not be appropriate (see Methods section 4.4.1). We found later that there were strong pleiotropic genetic effects for the Social-Emotional composite scale, the Adaptive scale, and the limiting pre-natal head circumference. A number of other phenotype dependencies were observed, and overall there was a clear relationship between robust growth and the Bayley phenotypes at age two. In our view it is best to consider these dependencies not as a collection of pairwise effects, but as a network of interdependencies implicating relationships among growth and Bayley phenotypes. We will address the network properties further when we consider regulatory effects implicated by our analysis.

**Table 1:** Significant dependencies among neurological phenotypes (Bayley) and growth parameters (Growth) with 2-way (top sub-table) and 3-way effects (middle and bottom sub-tables) were observed.

Bayley	Growth / Bayley	Growth	P-values
Cognitive	*alpha*		1.76E-05
Adaptive	*lambda*		3.29E-04
Social-emotional	*alpha*		4.7E-04
Language	*linf*	*alpha*	2.7E-04
Social-emotional	*linf*	*alpha*	1.293E-03
Adaptive	*linf*	*alpha*	1.905E-03
Cognitive	*linf*	*alpha*	2.257E-03
Adaptive	*alpha*	*lambda*	2.416E-03
Social-emotional	Adaptive	*linf*	2.22E-06
Social-emotional	Adaptive	*lambda*	3.97E-06
Language	ITSEA	*linf*	5.69E-06
Adaptive	CBCL	*lambda*	7.96E-06
Language	Social-emotional	*alpha*	8.87E-06
Language	Adaptive	*alpha*	1.02E-05

Note that the change in statistical significance after multiple hypothesis correction here is small. The composite Cognitive Bayley scale score and the growth rate parameter *alpha* showed the strongest 2-way dependencies, as measured by the p-value (described in Methods section 4.4.) There was a clear relationship between robust head growth and the Bayley phenotypes for both 2-way and 3-way dependencies. With the exception of the two shown in the bottom sub-table, the other child-specific neurological phenotype data such as Infant Toddler Social Emotional Assessment (ITSEA) and Child Behavior Check List (CBCL) were observed in 3-way dependency with weaker significance levels (p-values > 10^−4^) and are not reported here.

These results support the idea that there are significant dependencies between fetal and early childhood growth and neurological development that should be investigated further and suggest a strong biological connection between early growth and the development of the brain. This suggests that in order to explore the biological sources of the dependence, genetic effects on both of these phenotype classes should be examined.

### 2.2. Pairwise genetic relationships with neurological and growth variables

**Fig 1:**
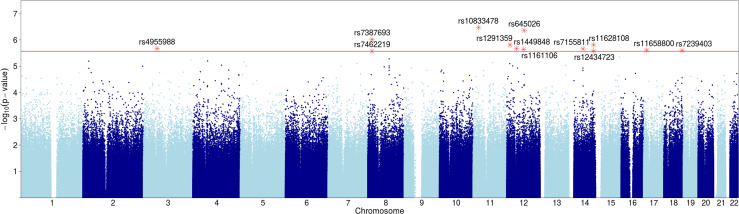
Manhattan plot of SNPs with Bayley phenotype dependence. Y axis shows p-values (negative log scale) of pairwise dependence of SNPs with Bayley phenotypes at 24 months (see Table 2). SNPs with p-value<2.7x10^-6^ (red line) are highlighted and labeled.

To explore the genetic relationships, we first examined the mutual information scores (see Methods section 4.3) between the 495,719 SNPs (a subset of 557,070 SNPs after preprocessing) and the five composite Bayley phenotypes for 433 subjects. The details of the acquisition of the Bayley phenotypes are provided in [16]. The subjects and the SNPs were those without any missing data values (see Methods section 4.2.4). The pairwise analysis shown in Table 2 reports permutation-based p-values as described in the Methods section 4.4.1 (only genetic effects with p-values better than 2.7x10^-6^ are shown). The collective conclusion derived from the number and nature of the implicated genes is that there are significant genetic influences on the neurodevelopmental phenotypes. We flagged these loci for further analysis (Fig 1). The genetic effects of one of these variants (NELL1) is shown in Fig 8A. The possible confounding effects of the different ethnicity were calculated as well. While a few SNPs have significant confounding effects, most do not. These results and calculations are discussed in Methods section 4.4.2.

**Table 2:** Significant SNPs associated with Bayley phenotypes using two-way dependency measures (mutual information, MI).

Bayley phenotype	SNP	Gene	MI	p-value
Motor	rs10833478	NELL1	0.0843	3.409E-07
Motor	rs645026	YEATS4	0.0832	4.398E-07
Adaptive	rs7387693	MTMR7	0.0847	9.542E-07
Adaptive	rs1291359	HTR7P1	0.0829	1.545E-06
Social-Emotional	rs11628108	C14orf177	0.0826	1.563E-06
Adaptive	rs4955988	CACNA2D3	0.0817	2.134E-06
Motor	rs1449848	CPNE8	0.0772	2.156E-06
Motor	rs7155811	TMEM260	0.0772	2.165E-06
Social-Emotional	rs1161106	LOC100507175	0.0811	2.284E-06
Language	rs11658800	ELAC2	0.0803	2.475E-06
Language	rs7239403	SMIM21	0.0803	2.514E-06
Social-Emotional	rs12434723	C14orf177	0.0805	2.608E-06
Adaptive	rs7462219	MTMR7	0.0807	2.667E-06

SNPs are ordered by p-values for the mutual information (see Methods section 4.3). Note that the positions of the SNP’s are indicated for the human genome build hg19. Note also that rs7239403 is closest to the non-coding RNA gene LINC01898, and SMIM21 is the closest protein-coding gene. The loci at MTMR7 are the only pair of variants in the same gene. These MI values are not adjusted for ethnicity confounding effects (see section 4.4.2).

Although in studies aimed at identifying causative SNPs, as is typical in GWAS, the p-value cutoff for significant SNPs is typically 5x10^-8^, driven largely by considerations of multiple hypothesis testing. The majority of our SNPs fall short of this cutoff and only two are better. This cutoff, however, has been shown to be very stringent, not taking into account correlations between variables (which we have in abundance among both SNPs and phenotypes), and is specifically meant to assure significance for causal SNPs [17–22]. In this paper we argued against applying this cutoff, or performing other common corrections for multiple hypothesis testing, since our goal is not to search for causal SNPs, but to detect a set of biologically relevant SNPs that may be statistically weaker on their own, but together can implicate pathways and processes of growth and neurological development. We therefore decided to use a higher p-value cutoff to allow for SNPs with weaker signals in this population to be collected for our downstream knowledge-based analysis. Since Bayley and Growth phenotypes are of different type (categorical vs numerical), we used two different cutoffs for selecting associated SNPs. We used 2.7x10^-6^ as a p-value cutoff for SNPs associated with Bayley phenotypes and 8x10^-6^ as a cutoff for growth associated SNPs (see Methods section). Tables 2 and 3 show the corresponding Bayley and Growth associated SNPs. The detailed descriptions of these considerations and methods are found in the Methods section.

**Table 3:** Significant SNPs associated with each of growth phenotypes (*linf*, *lambda* and *alpha*) using two-way dependency measures (mutual information, MI).

Growth parameter	SNP	Gene	MI	p-value
Linf	rs12734338	PPP1R12B	0.1130	2.229E-09
Lambda	rs6672510	PLD5	0.0316	3.120E-08
Linf	rs7071157	PFKFB3	0.0276	4.079E-07
Linf	rs6710428	CERS6	0.0264	8.826E-07
Lambda	rs4793500	CASC17	0.0259	1.163E-06
Alpha	rs9691259	IGFBP3	0.0258	1.299E-06
Linf	rs6570627	UTRN	0.0250	2.202E-06
Alpha	rs7101173	MIR6072	0.0248	2.543E-06
Linf	rs6884117	C5orf22	0.0241	3.858E-06
Lambda	rs564266	NTM	0.0241	4.054E-06
Lambda	rs7075547	LRRTM3	0.0237	5.153E-06
Lambda	rs773024	OSTF1	0.0236	5.530E-06
Linf	rs373680	FBXO33	0.0232	6.936E-06
Lambda	rs154444	ZNF608	0.0232	7.081E-06
Linf	rs7981995	DACH1	0.0232	7.181E-06
Alpha	rs10196354	ERBB4	0.0232	7.228E-06

The SNPs are ordered by p-value of the unadjusted mutual information (see Methods section 4.3) and the 16 loci that have p-value better than 8x10^-6^ are shown. For convenience the nearest gene to the SNP is indicated, even when there is a significant distance between them. Note that none of these SNPs are in the coding regions.

Similarly, we examined the mutual information scores between the 448,658 SNPs and the three growth parameters for 1053 subjects. The subjects and the SNPs were those without any missing data values (see Methods section 4.2.4). Table 3 shows the pairwise genetic effects with permutation-based p-values better than 8x10^-6^.

**Fig 2:**
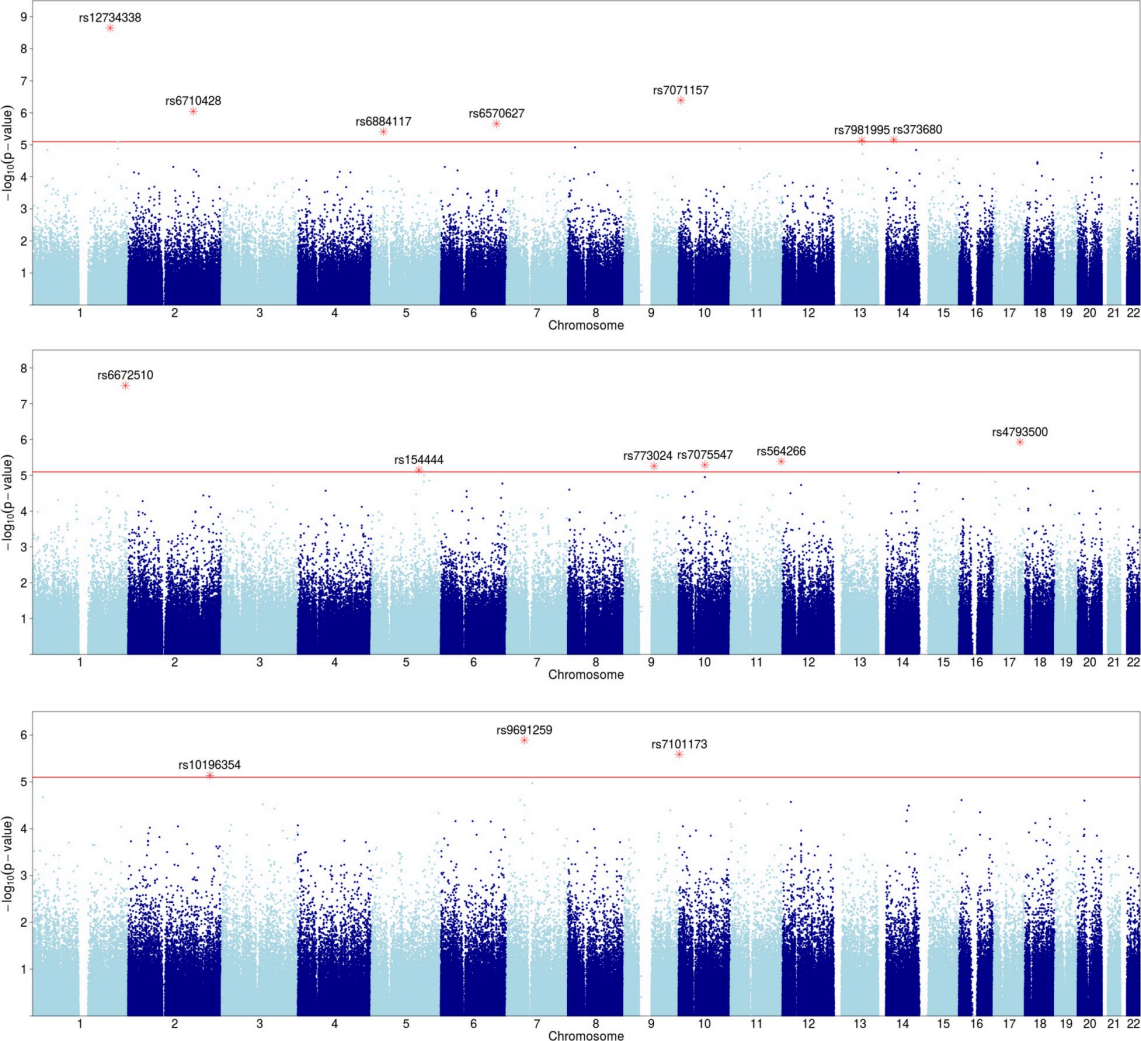
Manhattan plot of SNPs with growth parameter dependence. Y-axis shows p-values (negative log scale) of pairwise dependence of SNPs with three growth variables: **a**) *linf*, **b**) *lambda*, and **c**) *alpha*. SNPs with p-value<8x10^-6^ (red line) are highlighted and labeled.

These loci were associated with the three growth parameters, *linf*, *lambda* and *alpha*, considered as phenotypes (Table 3, Fig 2). Two loci showed notably strong effects (rs12734338 near PPP1R12B gene, rs6672510 near PLD5 gene). The former is a protein phosphatase subunit, which is implicated as the most significant celiac disease risk locus outside of the HLA region. This intronic SNP, rs12734338, was reported specifically for the Celiac risk effect [23]. The SNP rs9691259, with the highest score for *alpha* dependence, is notable since it is located near genes IGFBP3 and IGFBP1. Gene IGFBP3 produces insulin-like growth factor binding protein 3 directly involved in growth pathways, affecting growth factor stabilities, and also released by astrocytes in the brain [24]. Furthermore, rs9691259 is located between the 5’ end of IGFBP3 and the closest known enhancer (at coordinate 46,515,654 of genome build hg19). Thus, a regulatory effect is a reasonable conjecture for this genetic association.

### 2.3. Pleiotropic effects: Genetic locus dependence with pairs of neurological and growth variables

**Fig 3:**
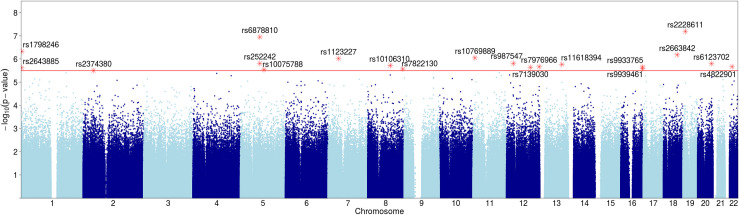
The Manhattan plots show significant SNPs for pleiotropy with growth parameters and Bayley phenotypes. Y-axis shows p-values (negative log scale) of three-way dependencies of SNPs, Bayley phenotypes, and each of the three Growth parameters: **a**) linf, **b**) lambda, and **c**) alpha. SNPs with p-value<3.2x10-6 (red line) are highlighted and labeled.

We used the three-way dependence method to discover pleiotropic genetic variants that were simultaneously interdependent with two phenotype variables, one each from the neurological and growth phenotype sets. The genetic variants in these three-way dependencies are not discovered by any pairwise dependence (see Methods section 4.3). For the three-way dependency calculation, we used 495,719 SNPs (without missing data), five composite Bayley phenotypes (Adaptive, Cognitive, Social-emotional, Motor and Language), and three growth model phenotypes (*linf*, *lambda*, and *alpha*) measured for 428 of 1073 subjects (without any missing values). This calculation identified 53 SNPs with candidate dependency for both neurological and growth phenotypes (Table 4, Fig 3). The locus with the most significant dependency is the SNP in the RAB11FIP4 gene, a highly brain specific gene (p-value of 2x10^-8^). This locus is associated with growth phenotype *lambda* and Bayley phenotype Adaptive (Table 4 and Fig 3) and is contiguous to the NF1 gene (neurofibromatosis), and therefore implicated in growth in the neural system. This variant is in an intron. The next most significant locus is within the DNMT1 gene (DNA methyl transferase 1, with a p-value of 6.5x10^-8^). This is a synonymous variant in an exon. Finally, the next locus is intronic to LHFPL2 and near ARSB (p-value 1.1x10^-7^), both brain-expressed genes. LHFPL2 has been reported to affect Parkinson’s and Alzheimer’s risk [25, 26].

**Table 4:** Statistically significant pleiotropic SNPs dependent with growth phenotypes (*linf*, *lambda* and *alpha*) and Bayley phenotypes using three-way dependency.

Chr	DBSNP	Nearest Gene	Major	Minor	Bayley Phenotype	Growth Phenotype	P-Val
17	rs178850	RAB11FIP4	C	T	Adaptive	*lambda*	2.02E-08
19	rs2228611	DNMT1	C	A	Adaptive	*alpha*	6.46E-08
5	rs6878810	LHFPL2	T	G	Social-Emotional	*alpha*	1.13E-07
16	rs9933359	LINC01082	C	T	Adaptive	*lambda*	2.26E-07
1	rs584297	LOC105373115	A	G	Adaptive	*lambda*	3.15E-07
1	rs525410	LAMC2	C	T	Social-Emotional	*lambda*	4.56E-07
1	rs1798246	PRDM16	A	G	Adaptive	*alpha*	4.68E-07
4	rs5020219	ANKRD17	T	C	Adaptive	*lambda*	5.97E-07
18	rs2663842	ATP8B1	C	A	Adaptive	*alpha*	6.62E-07
14	rs12587898	IFI27L1	G	T	Adaptive	*lambda*	6.70E-07
12	rs4763682	PRB4	A	C	Adaptive	*lambda*	6.70E-07
6	rs2064317	TULP1	A	G	Adaptive	*linf*	7.00E-07
15	rs936214	PAK6	C	T	Language	*linf*	7.54E-07
1	rs12030971	DEPDC1-AS1	A	G	Language	*linf*	7.91E-07
8	rs11787410	CSMD1	G	A	Adaptive	*lambda*	8.84E-07
11	rs10769889	LMO1	T	G	Social-Emotional	*alpha*	8.88E-07
7	rs1123227	LINC01448	G	A	Social-Emotional	*alpha*	9.56E-07
16	rs4843851	IRF8	A	C	Adaptive	*lambda*	1.02E-06
9	rs10869192	PIP5K1B	G	A	Adaptive	*linf*	1.04E-06
3	rs1131364	FETUB	C	A	Adaptive	*lambda*	1.07E-06
1	rs4846400	ESRRG	T	C	Adaptive	*linf*	1.16E-06
9	rs4741967	SLC1A1	T	C	Social-Emotional	*linf*	1.21E-06
22	rs5751491	LINC01639	A	G	Adaptive	*lambda*	1.32E-06
2	rs2164807	ATOH8	A	G	Adaptive	*linf*	1.39E-06
12	rs11608306	PRB2	A	G	Adaptive	*lambda*	1.50E-06
11	rs11224253	JRKL-AS1	C	T	Language	*linf*	1.51E-06
2	rs4668039	STK39	C	T	Adaptive	*lambda*	1.53E-06
9	rs9918994	LINC00587	T	C	Adaptive	*lambda*	1.54E-06
5	rs252242	LHFPL2	A	G	Social-Emotional	*alpha*	1.54E-06
12	rs987547	PPFIBP1	G	T	Social-Emotional	*alpha*	1.56E-06
20	rs6123702	CTCFL	C	T	Adaptive	*alpha*	1.60E-06
16	rs2938729	CDH8	G	T	Language	*linf*	1.61E-06
13	rs11618394	LINC00397	T	C	Adaptive	*alpha*	1.71E-06
10	rs10824914	FAM21EP	T	C	Adaptive	*lambda*	1.80E-06
8	rs10106310	LINC00534	C	A	Social-Emotional	*alpha*	1.92E-06
9	rs2150696	TYRP1	T	C	Language	*linf*	2.01E-06
22	rs4822901	LOC105372981	C	T	Social-Emotional	*alpha*	2.12E-06
9	rs871981	TYRP1	C	A	Adaptive	*linf*	2.13E-06
12	rs7976966	RIMBP2	C	A	Adaptive	*alpha*	2.15E-06
16	rs9933765	LOC101928737	G	A	Social-Emotional	*alpha*	2.17E-06
9	rs7029138	LINC00587	G	T	Adaptive	*lambda*	2.19E-06
12	rs7139030	NTN4	A	C	Social-Emotional	*alpha*	2.27E-06
5	rs770172	FBXL17	C	T	Social-Emotional	*linf*	2.34E-06
1	rs2643885	SKI	A	G	Adaptive	*alpha*	2.42E-06
12	rs4965006	PUS1	T	G	Language	*linf*	2.43E-06
16	rs9939461	LOC101928737	T	C	Social-Emotional	*alpha*	2.60E-06
4	rs2725270	ABCG2	T	C	Adaptive	*lambda*	2.66E-06
8	rs7822130	TRAPPC9	G	A	Social-Emotional	*alpha*	2.67E-06
16	rs7200646	LOC146513	A	C	Adaptive	*lambda*	2.87E-06
18	rs7232905	BOD1L2	G	A	Adaptive	*lambda*	2.88E-06
5	rs10075788	GPR150	T	G	Language	*alpha*	2.89E-06
2	rs2374380	LOC388942	C	T	Language	*alpha*	3.13E-06
3	rs2310229	ACPP	T	C	Adaptive	*linf*	3.16E-06

All 53 loci have a p-value better than 3.2x10^-6^. These results were obtained based on 428 subjects. The SNPs are ordered by p-values.

Because our three-way analysis uses the symmetric measure Delta3, which is the product of three factors, the asymmetric Deltas corresponding to each variable (see Methods section 4.3), it is not possible to determine which variable dependencies dominate. In order to capture dependencies that have only one or two large factors that might not be seen by the symmetric Delta3, we also examined each individual factor. These measures are specific for each variable (Δ_1_ for growth phenotypes, Δ_2_ for Bayley phenotypes, and Δ_3_ for SNPs). For the asymmetric Delta analysis the same five Bayley phenotypes, three growth model parameters, SNPs, and subjects were used. Each asymmetric Delta, Δ_1_, Δ_2_, and Δ_3_ identified 77 SNPs (S1 Table), 106 SNPs (S2 Table), and 117 SNPs (S3 Table), respectively, but with higher p-values. These analyses uncovered only a couple of additional loci (TRANK1, for example) suggesting that most of the collective dependencies detected by the symmetric delta are relatively balanced in their phenotype pleiotropies.

### 2.4. X and Y SNPs

If analyzed together with other SNPs, the X and Y SNPs overwhelm the statistical signal due to the genotype patterns distinguished by sex. As a result, the dependencies for the X and Y chromosomes were assessed separately and the subjects separated by sex. The results for all combinations of phenotypes and gender are listed in Table 5. The subjects for each analysis, and the preprocessing for this analysis are shown in S5 Table. The X-linked DMD gene (dystrophin) is a notable locus with 2 SNPs implicated by the Delta3 score for females. Note that the dystrophin gene has been previously reported to affect brain development [27]. Note that the number of subjects here was considerably smaller, after separating them by gender and removing subjects with missing data, resulting in a substantial loss of statistical power. With p-values less than 1.5x10^-5^ some of these are not very significant, but we include them here as candidates for potentially important pathways.

**Table 5:** Loci on X and/or Y chromosomes with p-values < 1.5x10^-5^.

Chr	Gender	DBSNP	Nearest Gene	Major	Minor	Bayley Phenotype	Growth Phenotype	P-Val	N
XY	Male	rs5949162	LOC107985677	G	A	Social-emotional	-	1.92E-07	227
XY	Male	rs306875	SPRY3	T	G	Adaptive	*linf*	1.15E-06	223
X	Female	rs5972504	DMD	T	C	Adaptive	*alpha*	5.64E-06	205
XY	Male	rs7054955	Intergenic	T	C	Adaptive	*linf*	8.62E-06	223
XY	Male	rs28562204	DHRSX	C	A	-	*lambda*	1.08E-05	551
X	Female	rs989011	GLRA2	T	C	-	*lambda*	1.37E-05	502
X	Female	rs2445644	DMD	G	T	Adaptive	*alpha*	1.45E-05	205
XY	Male	rs311043	CD99	T	G	Language	*linf*	1.46E-05	223

The SNPs are listed according to p-values. Note that this table includes both pairwise and 3-way dependencies, which are indicated by the presence of one or two phenotypes (indicated in the labeled columns). The numbers of subjects used are different because dependencies were computed separately for males and females, and the missing values were different in each case.

The two loci with the lowest p-values lie in the pseudo-autosomal regions Par1 and Par2 (at the ends of the X chromosome) respectively. The first is a gene of unknown function, however it is located over 40kbps from the implicated SNP. The second locus, the *sprouty* gene locus (SPRY3), implicated in males, is a gene reported to be involved in placental development [28]. Note that the two phenotypes in the dependency with SPRY3 are *linf*, related to head circumference, and Adaptive, a composite Bayley phenotype. The best 10 SNPs with respect to p-values in each phenotype category are listed in S4 Table.

### 2.5. Linkage disequilibrium SNPs

Recall that a large number of the original 933,886 SNPs with high mutual information between each other were removed to reduce redundancy before conducting the dependency analysis. To find additional SNPs potentially implicating other candidate genes, we searched for all possible SNPs in Linkage Disequilibrium (LD) with 82 SNPs previously identified using two-way and three-way dependency analysis (the SNPs in Tables 2–4). The LD was calculated for the same sets of subjects used in the corresponding dependency analyses (for details see Methods section 4.2.1). Although we identified 17 LD SNPs (section of S7 Table), they provided no new information about other potential genes that might affect the phenotypes (they were either within the same gene intron/exon or in the same intergenic region). Hence, the disequilibrium, while strong in many cases, did not add to our list of potential biological influences.

### 2.6. Gene interaction

The genetic dependencies reported in the previous sections are pairwise associations or pleiotropic effect variants. We expect that there may also be interactions involving multiple variants that contribute to the overall dependencies. Since the three-way measure can assess two variant effects on a phenotype, we calculated the interaction between each locus already implicated above and all other variants. For this calculation, 39 single locus effect SNPs that have been noted in neurological development or growth (see Tables 8 and 9) were combined with 495,718 other SNPs for each phenotype. The p-values for these measures were calculated using the same permutation methods as for the single locus effects. This resulted in the detection of interactions between loci detected in pairwise dependencies and loci not seen with any significant other dependence, as presented in Table 6.

**Table 6:** Locus interaction effects detected for notable loci exhibiting single locus effects.

SNP 1	Coord 1	SNP 2	Coord 2	Gene 1	Gene 2	P-value
rs2228611	19_10267077	rs10424964	19_10327812	DNMT1	S1PR2 (-)	5.32E-08
rs1131364	3_186370333	exm2249408	10_87772933	FETUB	GRID1 (-)	6.22E-07
rs1291359	12_13157267	rs2271025	16_66951783	HTR7P1	CDH16 (-)	4.91E-08
rs1291359	12_13157267	rs9374553	6_115937666	HTR7P1	FRK (-)	1.01E-07
rs1291359	12_13157267	rs10873367	14_86054406	HTR7P1	FLRT2 (+)	1.63E-07
rs1291359	12_13157267	rs11100377	4_162530770	HTR7P1	FSTL5 (-)	1.87E-07
rs1291359	12_13157267	rs7232315	18_55566232	HTR7P1	ATP8B1 (-)	1.90E-07
rs1291359	12_13157267	rs7552143	1_58260994	HTR7P1	DAB1 (-)	2.72E-07
rs1291359	12_13157267	rs220172	21_43556691	HTR7P1	UMODL1 (+)	2.93E-07
rs1291359	12_13157267	rs731957	16_85492882	HTR7P1	GSE1 (+)	2.95E-07
rs1291359	12_13157267	rs472771	1_48556821	HTR7P1	SKINTL (-)	3.52E-07
rs5020219	4_74036166	rs1381014	4_73862030	ANKRD17	COX18 (-)	8.23E-08
rs5020219	4_74036166	rs7666763	4_73858464	ANKRD17	COX18 (-)	8.06E-07
rs525410	1_183176430	rs13374873	1_30280472	LAMC2	LOC101929406	2.18E-07
rs936214	15_40565705	rs11903255	2_167464366	PAK6	SCN7A (-)	1.09E-07
rs936214	15_40565705	rs17025241	3_88053396	PAK6	HTR1F (+)	6.88E-07
rs987547	12_27715010	rs1436125	12_96299676	PPFIBP1	CCDC38 (-)	1.86E-07
rs178850	17_29759235	rs6665385	1_176114487	RAB11FIP4	RFWD2 (COP1)	2.22E-07
rs178850	17_29759235	rs1993451	12_125137009	RAB11FIP4	SCARB1 (-)	2.31E-07
rs178850	17_29759235	rs704834	1_176189141	RAB11FIP4	PAPPA2 (+)	2.34E-07
rs4741967	9_4374278	rs17741020	9_4359689	SLC1A1	SLC1A1 (+)	3.77E-07
rs6123702	20_56055633	rs2075755	19_6422888	CTCFL	KHSRP (-)	7.57E-08
rs10869192	9_71280103	rs715521	22_48454426	PIP5K1B	LOC284930 (+)	1.19E-07

Gene 1 here indicates a locus that has been noted for several reasons: expression profiles, brain or growth specific known effects, or low p-values in a single locus effect (see Tables 8 and 9). The coordinates are for genome build hg19. The p-values are calculated for the three-way interaction measure (Delta3 for two SNPs, one of which is a and the single locus effect SNP).

There are a number of notable interacting pairs here, for example, the variant at the sphingosine-1-phosphate receptor 2 gene shows significant interaction with DNMT1 and both of these genes are strongly expressed in the placenta. The locus at HTR7P1 shows interactions with a diverse range of other loci, on eight different chromosomes. It is clearly an interaction hub of some kind. The significance of multiple interactions, including the RAB11FIP4 and PAK6 loci is currently unclear, but intriguing.

### 2.7. Functional genomic analysis

#### 2.7.1. Variant annotation

**Fig 4:**
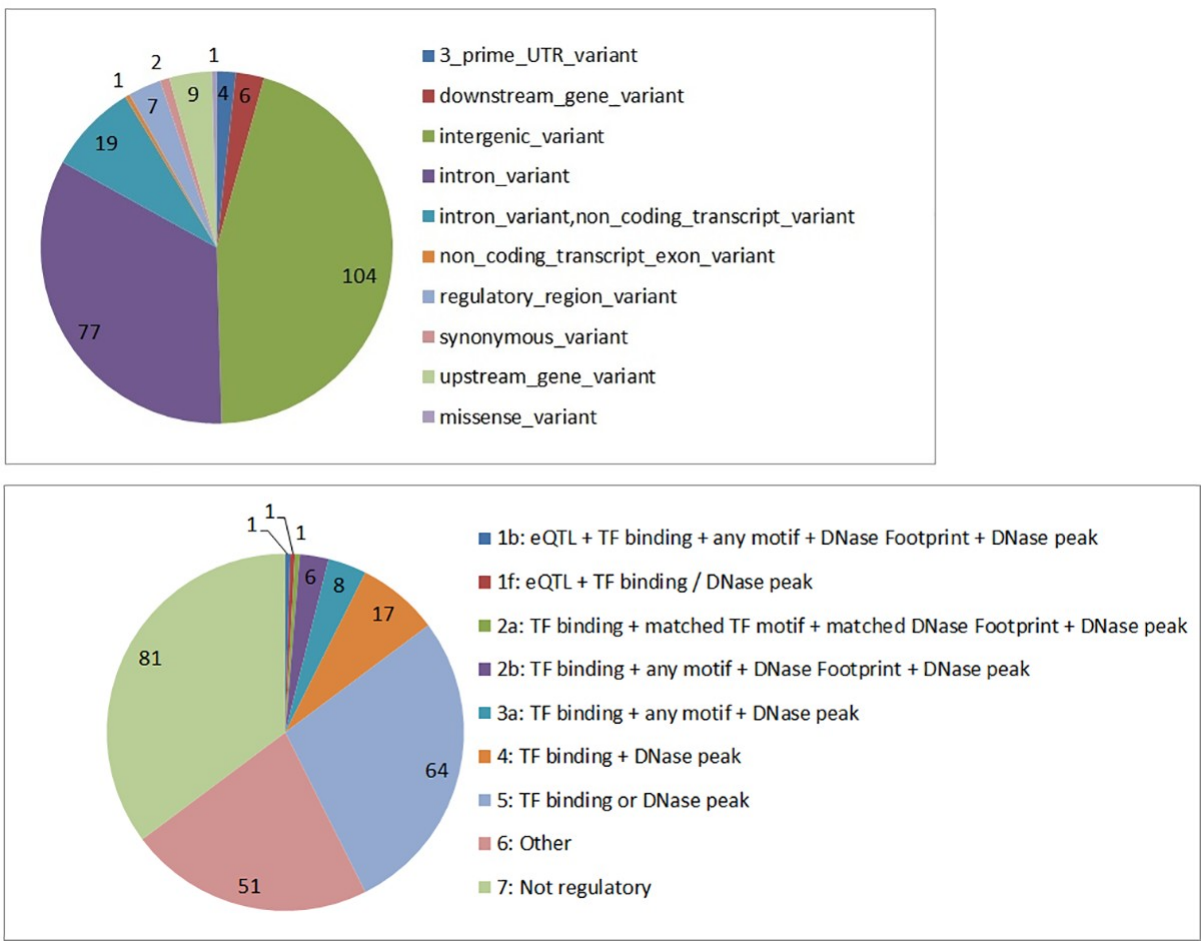
Distribution of functional consequence using VEP annotation tool and RegulomeDB scores for SNPs linked to neurological and growth phenotypes identified through two-way (mutual information) and three-way (Delta 3) dependency analysis. **a**) Distribution of candidate SNPs across the functional locations based on VEP annotation. Most of the SNPs are located in non-coding locations, i.e., intronic and intergenic regions of the genome. **b**) The RegulomeDB score for the candidate SNPs. The lower the score, the more likely it is that a SNP has a regulatory function. eQTL = expression Quantitative Trait Loci; TF = Transcription Factor.

To investigate the potential biological interactions implicated by the genetic dependencies, we integrated the candidate sets of SNPs identified by two-way and three-way analysis (from Tables 2–4). The list contains 230 unique SNPs after removing 3 SNPs lacking mapping information. We re-annotated the candidate variants based on their location in the genome using Variant Effect Predictor (VEP, https://www.ensembl.org/vep). Functional annotation of these 230 SNPs showed that majority were non-coding and located either in the intergenic or intronic regions of the genome (Fig 4A). There were two neutral coding SNPs and one missense SNP (rs2064317) located in the coding region of TULP1 gene.

#### 2.7.2. Regulatory functional analysis

The majority of the SNPs identified in GWAS studies to date are located in the noncoding regions of the genome, and even though they may have been implicated simply because of their linkage disequilibrium with a causative SNP, they are equally likely to point to regulatory elements [29, 30]. As most of the SNPs we identified are also located in non-coding regions of the genome, it is likely that there are some regulatory effects. To carry out this analysis we used RegulomeDB [31] (http://www.regulomedb.org/). Of the 230 SNPs, 148 SNPs were scored as having potential regulatory effects (Fig 4B and S6 Table) with two known eQTL SNPs, rs2164807 in the regulatory region of ATOH8 gene, and rs525410 in the intronic region of LAMC2 gene.

**Fig 5:**
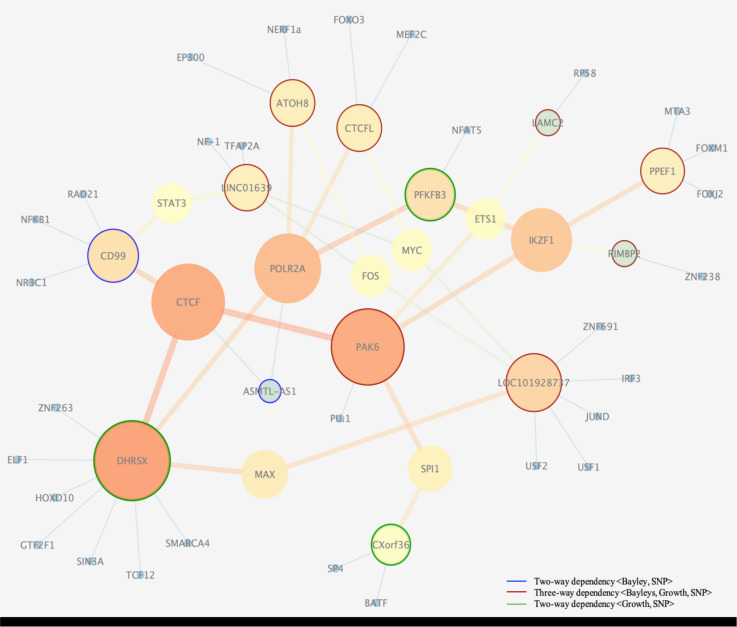
Regulatory interaction network. Depicted are interactions of transcription factors connected with regulatory SNPs (noted by their nearest gene). Clustering and visualization of the network was carried out using Cytoscape v.3.3.0 (undirected network and betweenness centrality statistics). The degree of nodes (the number of edges per node) is shown with their color, ranging from orange (the highest degree), to yellow, green, and then blue (the lowest degree). In addition, larger nodes correspond to hubs with higher degree. The edges with high betweenness centrality, whose removal would partition the network into connected subnetworks, are depicted by thick, orange lines. The small blue nodes are additional factors connected to the dependent loci. “Orphan genes” (unconnected nodes) are not shown. Nodes with blue, green, and red rings correspond to loci detected with two-way and three-way analysis (see the legend).

Furthermore, 17 SNPs were scored by RegulomeDB as having strong regulatory functions, indicated by the top 5 categories, namely 1b, 1f, 2a, 2b, and 3a (see Fig 4B for description). These SNPs together with their 69 transcription factor (TF) co-regulators (see S8 Table) are part of a regulatory network governing child’s growth and neurocognitive development. Regulatory genetic networks underlying a phenotype arise from regulatory SNPs affecting the transcription factor recognition sequences. To reconstruct a transcription regulatory network, we connected the SNPs (annotated here by their nearest genes) with common regulatory interactors/TFs as intermediate components, allowing for a connected sub-network (genes without any connections, were excluded). The network of regulatory interactors (Fig 5) connected 13 key regulators (SNPs in regulatory regions of ATOH8, CTCFL, LINC01639, CD99, PFKB3, LAMC2, PPEF1, RIMBP2, LOC101928738, CXorf36, PAK6, ASMTL-AS1, DHRSX) with 38 TF interactors. Of the 13 genes with regulatory SNPs in the network (Fig 5), PAK6 seems to be the central node in the network.

PAK6 identified through our three-way dependence analysis belongs to a group of p21-stimulated serine/threonine kinases, and is a key regulator of signal transduction pathways, cellular division regulation, gene transcription, cytoskeleton rearrangement and apoptosis. PAK6 protein expression profile points to highest expression in tissues such as skin, placenta, testis and cerebral cortex [32] (https://www.proteinatlas.org), while the RNA expression profile distinctly points to brain tissue specific expression including cerebral cortex and caudate (https://www.proteinatlas.org, http://gtexportal.org). In a study carried out by Nekrasova et al. [33], it was shown that PAK6 is highly expressed in the brain and PAK5/PAK6 double knockout mice exhibit several locomotor and behavioral deficits. Nekrasova and colleagues concluded that normal expression of these two proteins are required for normal level of activity, and for normal learning and memory, which suggests an important role of PAK6 in neurological and growth development.

#### 2.7.3. Gene expression profiling

In addition to reconstructing a transcription regulatory network using RegulomeDB, as shown in Fig 5, we analyzed tissue specific gene expression of genes and/or eQTLs associated with our integrated set of SNPs, using the Genotype-Tissue Expression (GTEx) database (http://gtexportal.org). Using GTEx we identified 56 SNPs, out of which 2 were previously detected by RegulomeDB (S6 Table). Moreover, several of these SNPs were also shown to have a modest effect on the expression of their associated genes in tissues such as skeletal muscle, tibial nerve and several brain tissues/regions (see Table 7).

**Table 7:** Three SNPs identified with tissue specific gene expression of their associated genes and /or eQTLs, using the Genotype-Tissue Expression (GTEx) database (http://gtexportal.org).

**SNP**	**Gene**	**Tissue**	**NES**
rs12587898	IFI27L1	Brain cortex	0.41
rs1179161	PNPLA4	Tibial nerve	0.42
“	“	Frontal cortex	0.21
“	“	Putamen	0.25
“	“	Spinal cord	0.36
“	“	Cortex	-0.39
“	“	Caudate	0.18
rs645026	YEATS4	Caudate	-0.45

The normalized effect size (NES) defined as the slope of the linear regression of the effect of the alternative (minor) allele relative to the reference (major) allele, based on hg19 reference genome [34].

#### 2.7.4. Application of functional mapping and annotation of genome-wide association studies (FUMA)

We combined functional annotation and gene mapping results using known biological databases to look for additional evidence about the key variants likely to play a part in neurological and growth development. For this purpose, the integrated set of SNPs was explored using the software package *Functional Mapping and Annotation of Genome-Wide Association Studies* (FUMA) [35–38] (http://fuma.ctglab.nl). FUMA has previously been used in several GWAS including studies of intelligence [39], neuroticism [40], and Parkinson’s disease [41].

We selected two particularly interesting lead risk loci, rs178850 and rs6672510, to analyze using FUMA. The first SNP, rs178850, has the best p-value (2x10^-8^) of those identified in a three-way dependency with Adaptive (Bayley phenotype) and *lambda* (growth phenotype). This SNP is particularly interesting as it is located in the intronic region of RAB11FIP4 gene on chromosome 17 and is also very close to NF1 and OMG, which is another brain-specific gene located within NF1. Thus this SNP could affect three brain genes. See S9 Fig for the details of the one-SNP three genes structure. All three of these genes are highly expressed in brain. GTEx data from 53 tissue types shows that RAB11FIP4 has the highest expression in all 13 brain tissues in normal samples, which highlights its potential role in neurological development and growth, and its neighbor, NF1, is expressed almost exclusively in the brain and nervous system.

The second interesting SNP, rs6672510, was identified in two-way dependency analysis with *lambda* (growth rate parameter phenotype) with a p-value of 3.12x10^-8^. This SNP is located in the intronic region of PLD5 gene on chromosome 1. Like in the previous example, it is also shown to be highly expressed in brain, though its associated phenotype is growth.

**Fig 6:**
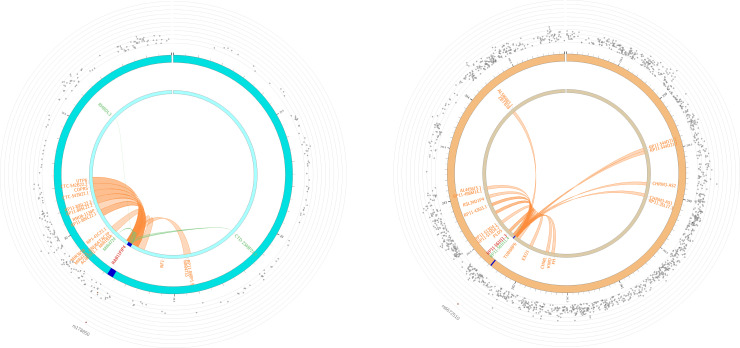
FUMA circular plots of chromatin interactions and eQTLs of lead SNPs. **a**) The plot of chromosome 17, showing the lead SNP, rs178850, of RAB11FIP4 gene and its interactors. **b**) The plot of chromosome 1, illustrating the second lead SNP, rs6672510, of PLD5 gene and its interactors. The outer ring (grey dots) shows the Manhattan plot of all the SNPs in the chromosome, with p < 0.05, and not in LD with the lead SNP. The lead SNPs are indicated with a red dot. Both inner rings indicate the chromosome, with the risk loci highlighted in blue. The links and labels indicate chromatin interactions (orange) and eQTLs (green). When the SNP is mapped by both chromatin interactions and eQTLs, as in the case of rs178850, it is highlighted as red.

FUMA generated circular plots (Fig 6), indicating positions of chromatin interactions and eQTLs of the two lead SNPs (see Methods section 4.5.5). In the case of RAB11FIP4, 21 genes were linked to the risk locus, three *via* eQTL mapping and 18 *via* chromatin interactions (Fig 6A). In the case of PLD5, 20 genes were linked through chromatin interactions and one through eQTL mapping (Fig 6B). While not all the genes identified here are relevant to neurological and growth development, they can serve to identify additional genes and regions that are not indicated by proximity to the genetic variants and could be used in future experimental studies. Specifically, four genes (UTP6, CTC-542B22.2, COPRS, RP11-848P1.5) and three eQTLs (MIR4724, CTD-2349P21.9, RHBDL3) linked to the lead SNP in the RAB11FIP4 gene have been shown to be highly expressed in several brain tissues (https://gtexportal.org). Similarly, five genes (AL590483.1, ZBTB18, EXO1, CHML, KMO) linked to the lead SNP in *PLD5* have been shown to have high expression levels in several regions of the brain. The presence of chromatin interactors with expression profiles in brain tissues similar to RAB11FIP4 and PLD5, both of which are highly brain-expressed genes, is therefore highly suggestive of their roles in a regulatory network of neurological development.

For more information obtained from FUMA and GTEx applied to our candidate set of genes see S7 Table. The analysis of our set of candidate genes showed differential expression in frontal cortex, hypothalamus, caudate, nucleus accumbens and putamen, all known for affecting cognitive and motor functions.

#### 2.7.5. Estimated effects of interacting SNPs

Since the visualization of two-variable dependence with genetic variants is straightforward it is interesting to examine the distribution of the phenotypes in the population of children. Here we show three diverse examples of results to illustrate the way in which the genetic variants in this cohort affect the phenotypes. It is clear that the distributions of these quantitative phenotypes are distinctly different. This effect is seen both for the spectrum of Bayley phenotypes in 24-month infants and in growth parameters. We illustrate the result for three phenotypes as a function of the variants labeled by their closest genes *NELL1* (composite Bayley phenotype, Motor), *PPP1R12B* (limit head size, *linf*), and *PLD5* (growth rate, *lambda*).

These genotype-specific profiles are interesting in several ways: the NELL1 stratification suggests that the effect on the Bayley phenotype of the minor allele is recessive to the major allele. For the PPP1R12B profile, the effect of a single minor allele seems to sharply affect the head size distribution. The fact that there are no observed homozygous minor genotypes at all at this position raises the question of whether the shift to smaller head size of the heterozygote may be highly detrimental in the homozygous state. In the third example of PLD5, the major allele appears to be partially recessive to the minor allele, which reduces the average growth rate.

**Fig 7:**
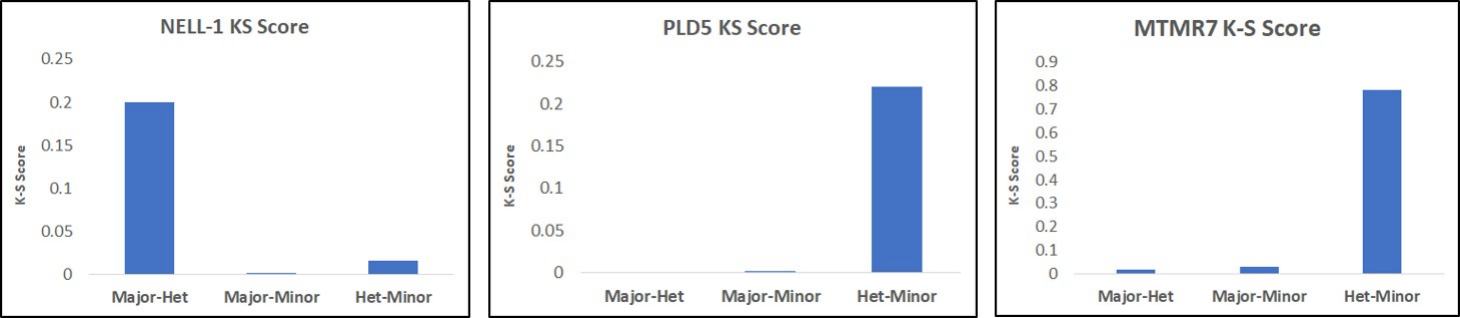
The Kolmogorov-Smirnov scores for the genetically stratified phenotype distributions (two shown in Fig 8). The scores, indicating the similarity between the distributions, show the dominance of the major allele for NELL1 and the dominance of the minor allele for PLD5, and MTMR7 (not shown in Fig 8).

Another, more quantitative, way to compare the distributions for specific SNP genotypes is to use a Chi-square or Kolmogorov-Smirnov (K-S) test, which provides a useful way to visualize pleiotropic dependencies. To illustrate its use, we show K-S tests for pairwise dependencies for NELL1, PLD5 and MTMR7 (see Fig 7). Here the K-S score indicates the p-value of testing the hypothesis that the distributions are the same.

**Fig 8:**
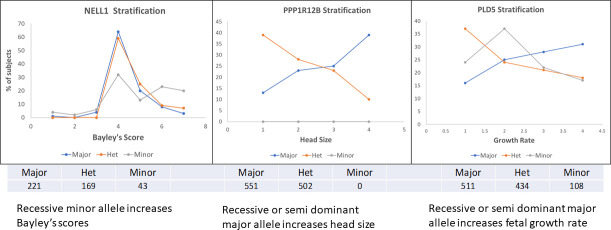
The distributions of phenotypes by genotype for three of the pairwise genetic variant effects. The numbers of subjects with each genotype are shown under each panel. **a**. NELL1 shows a distribution that suggests a strong dominance of the major allele for the Bayley scale score distribution. **b**. PPP1R12B shows a diametrically opposite distribution between the homozygote and heterozygote. **c**. PLD5 shows the same as in b) but with a distinct homozygous minor distribution.

**Fig 9:**
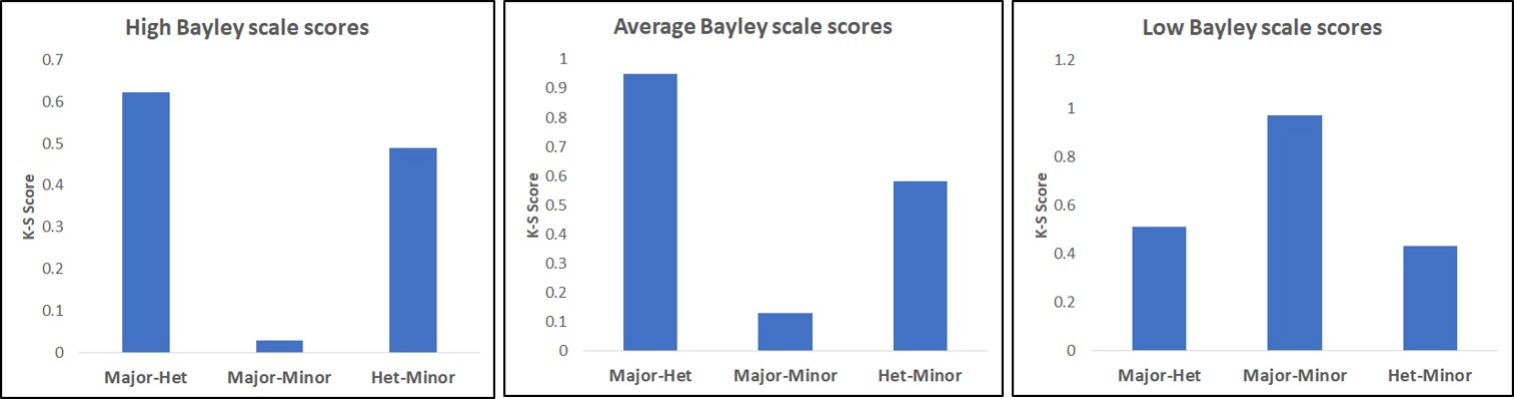
The Kolmogorov-Smirnov (K-S) test of similarity of distributions for the growth parameter phenotype (*linf*) genetically stratified according to PAK6 genotype (similarly to Fig 7). The K-S test is shown for different values of Bayley scale phenotype (Composite language score at 24 months).

Using the same measure, we can now visualize the pleiotropy by looking at the similarities and differences between phenotype distributions for different values of the second phenotype, as shown in Fig 9 for the pleiotropic variant in the PAK6 gene.

Clearly profiles of the growth (*linf*, the head circumference at birth) phenotype distribution for subjects with high and average values of Bayley phenotypes (Language score at 24 months) are similar, but the profile of the growth parameter for low values of Bayley phenotypes is distinctly different. While it is often difficult to visualize the complex three-variable dependencies inherent in pleiotropic genetic effects, these measures of similarity seem to provide useful profiles.

## 3. Discussion

The finding of significant dependencies among the variables characterizing fetal and early childhood growth and those characterizing neurological development in the GUSTO project data led us to explore the genetic dependencies of these variables. The overall goal of this effort was to gain insight into the underlying biological mechanisms in healthy children and to implicate processes and pathways involved. In order to mobilize the results of the genetic analysis of this large data set into possible insights that point to mechanistic pathways and networks involved in these critical processes we analyzed and integrated the results in several ways.

We used our three-way dependence measure here to identify complex relationships, in this case pleiotropies, for the first time in human data. Considering that the cohort was not selected for any traits, and appeared to be normal, healthy children, the results were striking. First, we found several genetic dependencies of neurological development as indicated by the five different Composite Bayley scale scores at two years of age. Second, genetic dependencies of the fetal and early childhood growth parameters were also identified using parameters fit to growth data as phenotypes. The set of candidate genes identified using the pairwise measure (mutual information), with potential functions known to affect growth and brain development and function, included some intriguing candidates and were encouraging. We then looked for genetic effects on two phenotypes together, pleiotropic effects, using the three-way measure from our multivariable dependency method, and found another set of interesting candidates. Our information-based dependency measures confer the advantage of reduced sensitivity to undersampling relative to a model fitting approach, so that the number of subjects and the potential complexity of the dependencies in this work yield results that permutation tests suggest are significant.

The largely disjoint sets of SNPs in the three classes (affecting growth parameters, Bayley phenotypes, and both together) is perhaps surprising, since one might expect that a SNP affecting both neurological development and early growth should have a significant presence for two-way dependence for each class of phenotypes. As we have discussed in previous work on multiple dependencies [15], this is not necessarily the case. To further explore this disjoint effect, we looked at the two-way dependencies for each of the SNPs identified in the three-way analysis and confirmed that there were no significant two-way dependencies. The initial lists of results tell the full story. While the interpretation of this observation is unclear, it seems to indicate that the source of the three-way effects is largely distinct from the two-way effects.

The use of our three-variable dependency measure has been shown to yield a number of interesting results that could not be detected using only two-way methods [15], which has significant implications for the way in which human phenotype data are analyzed. Finding three-way effects that are distinct from any two-way effects represents a sharp shift of approach and should be considered in future studies.

Keep in mind here that our insights are based on attempted interpretation of the effects of SNPs that fall largely in intergenic regions and introns. While this means that we are attempting to implicate some genes by their proximity to the SNPs, we also have used regulatory data analysis, and gene expression profiles to attempt to pull pieces of the puzzle together.

The fetal and early childhood growth parameters and the neurological development show a pattern of dependency on one another, and the genetic effects on both classes of phenotypes that we see are striking. It is yet unclear what the most important biological pathways involved in these effects are, but it is intriguing that the patterns are rather consistent in the prevalence of brain-specific or brain-related genes. It is not surprising that SNPs near genes that are expressed in the brain and CNS are implicated in the neurological development, but this pattern is also present in the three-way dependencies with growth and neurological development. It is clear, perhaps not surprisingly, that the overall growth of the head circumference and the development of the infant brain are strongly coupled. It is probably worth further investigation to also determine the extent to which the growth of the early brain may be involved in regulating the overall growth of the fetus.

To explore the biological relevance of the candidate SNPs identified using our two-way and three-way dependency measures, we compiled the set of 230 variants, including LD SNPs and those located on the X and Y chromosomes. Functional annotation of the integrated set of SNPs showed that majority are in intronic and intergenic regions, so we examined the potential regulatory functions of this set using RegulomeDB and identified two eQTL SNPs, selected using three-way dependency analysis between neurological and growth phenotypes (S6 Table). The eQTL SNPs rs2164807 (p-value of 1.39E-06; identified through dependency between <Adaptive, *linf*, SNP>) and rs525410 (p-value of 4.56E-07; identified through <Social-Emotional, *lambda*, SNP>) are located in the regulatory regions of ATOH8, a transcription factor involved in nervous system development (GO:0007399) [42] and LAMC2 implicated in neurite outgrowth among other functions [43], respectively.

The many genes identified in this work represent the multiple pieces of evidence that can point to processes and pathways. While this integration is clearly at its outset, we can illustrate something of its value by a comparison of the p-values linking SNPs to Bayley phenotypes (either two- or three-way dependencies), relative levels of expression in the brain, placenta and other relevant information, and the attribution of effects of variants in genes on human phenotypes as recorded in the literature. To illustrate this kind of integration, we compiled two tables of relevant SNP variants that could be linked to neurological development. While it is somewhat artificial to separate growth and brain development in the presence of so many pleiotropic effects, we do so for simplicity. They should be considered together. Table 8 identifies the SNPs, the genes nearby or containing the SNPs, the expression levels and effects linked to brain and neurological development or linked to relevant human traits reported in the literature.

**Table 8:** Summary of neurological development variants.

Chr	rsID	Nearest Gene	Bayley Phenotype	Growth Phenotype	P-Val	N	Relevant Expression & effects of variants	Literature Reference
17	rs178850	RAB11FIP4	Adaptive	*lambda*	2.02E-08	428	Highly brain specific (7-10-fold higher than other tissues), affects neurofibroma growth, next to NF1 and OMG	Bartelt-Kirbach *et al*., 2009 [44]
1	rs6672510	PLD5	*	*lambda*	3.12E-08	1053	High expression in brain, then adrenal and ovary, previously associated with autism and multiple sclerosis.	Anney *et al*., 2010 [45], Baranzini *et al*., 2009 [46]
5	rs6878810	LHFPL2	Social-Emotional	*alpha*	1.13E-07	428	Genetic modifier of Parkinson's age of onset, new AD susceptibility locus	Hill-Burns *et al*., 2016 [25], Potkin *et al*., 2009 [26]
XY-PAR1	rs5949162	LOC107985677	Social-emotional	*	1.92E-07	227 (M)	high expression in brain and endometrium	
1	rs584297	LOC105373115, (near STUM)	Adaptive	*lambda*	3.15E-07	428	STUM codes for highly brain specific, mechanosensory protein	Desai *et al*., 2014 [47]
1	rs525410	LAMC2	Social-Emotional	*lambda*	4.56E-07	428	LAMC1 is just upstream, highly placenta specific, LAMC2 is near NMNAT2, brain specific, cognitive traits candidate	Bi, *et al*., 2017 [48], Sun *et al*., 2008 [43]
8	rs7387693	MTMR7	Adaptive	*	9.54E-07	433	Highly brain specific, candidate for Creutzfeldt-Jacob risk, smoking cessation	Sanchez-Juan *et al*., 2012 [49]
12	rs1291359	HTR7P1	Adaptive	*	1.55E-07	433	Serotonin receptor pseudogene, SNP is in promoter region of HEBP1 (Immune system related)	
8	rs11787410	CSMD1	Adaptive	*lambda*	8.84E-07	428	High brain and testis expression, linked to schizophrenia risk	Sakamoto *et al*., 2016 [50]
11	rs10769889	LMO1	Social-Emotional	*alpha*	8.88E-07	428	Pediatric neuroblastoma susceptibility candidate, expressed in adrenal, brain and skin	Oldridge *et al*., 2015 [51]
3	rs4955988	CACNA2D3	Adaptive	*	2.13E-06	433	highest expression in brain, next in adrenal, calcium channel subunit, role in regulating iron uptake	Baeza-Richer, Carlos *et al*. (2013) [52]
8	rs7462219	MTMR7	Adaptive	*	2.67E-06	433	Highly brain specific, candidate for Creutzfeldt-Jacob risk, smoking cessation	Sanchez-Juan *et al*., 2012 [49]
XY—PAR2	rs306875	SPRY3	Adaptive	*linf*	1.15E-06	223 (M)	Brain expression, Placental expression, autism candidate	Ning *et al*., 2015 [53]
9	rs4741967	SLC1A1	Social-Emotional	*linf*	1.21E-06	428	Glutamate transporter implicated in epilepsy, linked to OCD risk	Afshari *et al*., 2015 [54], Arnold, *et al*., 2006 [55]
22	rs5751491	LINC01639, just upstream of MPPED1	Adaptive	*lambda*	1.32E-06	428	Almost exclusively brain expressed, region linked to schizophrenia risk, bipolar disorder and brain malignancies	Chen *et al*., 2010 [56]
17	rs11658800	ELAC2	Language	*	2.48E-06	433	High expression in the brain tissues	
16	rs2938729	CDH8	Language	*linf*	1.61E-06	428	Highly brain-specific	
11	rs10833478	NELL1	Motor	*	3.41E-07	433	Brain and kidney specific, interacts with neural EGFL	Shen et al., 2016 [57]
14	rs7155811	TMEM260	Motor	*	2.17E-06	433	Implicated in pediatric neural development	Ta-Shma *et al*., 2017 [58]
3	rs7611656	TRANK1*	Adaptive	*lambda*	3.36E-06	428	High endometrial expression, bipolar candidate	Chen *et al*., 2013 [59], Ruderfer *et al*., 2014 [60]
14	rs11628108	C14orf177	Social-Emotional	*	1.56E-06	433	Testis expression, linked to late onset Alzheimer's	Kunkle *et al*., 2016 [61]
14	rs12434723	C14orf177	Social-Emotional	*	2.61E-06	433	Testis expression, linked to late onset Alzheimer's	Kunkle *et al*., 2016 [61]

Features of a collection of the genes linked to 22 SNPs that we have tied to the neurological traits, having p-values < 5x10^-6^ and either: high brain levels of expression or specificity of expression, or published phenotypic effects related to neurological functions in human studies. The notation (M) in the N column indicates that the dependency was determined for male subjects only. TRANK1, marked with an asterisk, falls just below the p-value threshold so does not appear in Table 4.

Similarly, we compiled a table of relevant SNP variants we identified that could be linked to growth in a broader sense and therefore could be directly relevant to fetal and early childhood development. Table 9 identifies 17 of these SNPs, the genes nearby or containing the SNPs, the expression levels and links to the literature.

**Table 9:** Summary of some variants linked to fetal and early childhood development.

Chr	rsID	Nearest Gene	Bayley Phenotype	Growth Phenotype	P-Val	N	Relevant Expression & effects of variants	Literature Reference
1	rs12734338	PPP1R12B	*	*linf*	2.23E-09	1053	protein phosphatase 1 regulatory subunit, expression in heart, skeletal muscle, brain and endometrium, link to celiac disease & asthma in children	Moorhead *et al*. 1998 [62], Okamoto *et al*. 2006 [63], Freidin and Polonikov, 2013 [64], Östensson *et al*. 2013 [23], Montén et al 2015 [65]
1	rs6672510	PLD5	*	*lambda*	3.12E-08	1053	High expression in brain, adrenal and ovary	Anney *et al*. 2010 [45], Baranzini *et al*. 2009 [46]
19	rs2228611	DNMT1	Adaptive	*alpha*	6.46E-08	428	DNA methyl-transferase—Highest expression in placenta, transcript level associates with placental weight	Mukhopadhyay *et al*. 2016 [66], Branco *et al*. 2016 [67]
10	rs7071157	PFKFB3	*	*linf*	4.08E-07	1053	High expression in skeletal muscles, regulates glycolysis & cyclin-dependent kinase 1 Links glucose metabolism to cell proliferation, involved in brain development (GO:0007420)	Kessler & Eschrich, 2001 [68]
1	rs525410	LAMC2	Social-Emotional	*lambda*	4.56E-07	428	Expressed in several fetal tissues and placenta	
1	rs1798246	PRDM16	Adaptive	*alpha*	4.68E-07	428	Linked to obesity, heart function and T2D	Pérez-Belmonte *et al*. 2017 [69]
4	rs5020219	ANKRD17	Adaptive	*lambda*	5.97E-07	428	widespread expression, interacts with cyclin-dependent kinase 2	
14	rs12587898	IFI27L1	Adaptive	*lambda*	6.70E-07	428	expression high in in testis, adrenal and ovary, linked to anthropometric traits (height, weight etc.)	
15	rs936214	PAK6	Language	*linf*	7.54E-07	428	High expression in brain tissues, Kinase involved in cell proliferation and adhesion, placental expression	
1	rs12030971	DEPDC1-AS1	Language	*linf*	7.91E-07	428	regulates mitotic progression, placental expression	Mi *et al*., 2015 [70]
7	rs1123227	LINC01448	Social-Emotional	*alpha*	9.56E-07	428	Placenta and testis specific expression	
7	rs9691259	IGFBP3	*	*alpha*	1.30E-06	1053	Prolongs half-life of IGFs, high expression in placenta. Low levels linked to aging and cell senescence	Hong and Kim, 2018 [71]
3	rs1131364	FETUB	Adaptive	*lambda*	1.07E-06	428	Liver-specific expression, linked to osteogenesis and bone resorption, regulation of the insulin and hepatocyte growth factor receptors	
1	rs4846400	ESRRG	Adaptive	*linf*	1.16E-06	428	estrogen-related receptor, regulates DNMT1, involved in bone formation and cell growth regulation	
2	rs2164807	ATOH8	Adaptive	*linf*	1.39E-06	428	Transcription factor, highly expressed in fat cells, linked to muscle regeneration	Güttsches *et al*., 2015 [72]
12	rs987547	PPFIBP1	Social-Emotional	*alpha*	1.56E-06	428	tyrosine-phosphatase interacting protein, high expression in heart and placenta	
20	rs6123702	CTCFL	Adaptive	*alpha*	1.60E-06	428	ZF transcription factor, insulator factor, spermatocyte-specific expression	

Features of a collection of genes linked to 17 SNPs that we have tied to the neurological traits, having p-values < 1.6x10^-6^. These had either: expression in the placenta or endometrium, or published phenotypic functions related to cell growth and selected other functions that could be connected to early development functions in human studies.

The X-linked gene, *DMD*, did not have a low enough p-value to be included in the above tables (1.5x10^-5^), but it is particularly relevant to brain and fetal and early childhood development, and should be kept in mind as a possible player in some cases. This is the gene mutated in Duchenne muscular dystrophy. The dystrophin protein provides a key part of an actin-binding, multifunctional unit, a complex that provides a key component of an astrocyte “foot” that engages neurons in the developing brain [20]. There is now clear evidence of developmental disturbances that result in neuropsychiatric abnormalities in children, particularly males with mutations in *DMD* [73]. Dystrophin is also widely expressed, and therefore likely is engaged in more functions than only in brain and muscle as part of the dystrophin associated complex. We should therefore consider the link to the DMD gene in this study as a pointer for future investigation.

We have explored the integration of the identified set of predicted regulatory SNPs (annotated by their nearby genes) in another way by constructing a regulatory network to find key genes and/or transcription factors potentially involved in neurological and growth development and evaluated their expression profile in normal tissues using GTEx database [34] (https://gtexportal.org). The transcription factor regulatory network constructed by RegulomeDB (Results section, Fig 5) points to key genes, most of which were identified through our three-way dependency measure. Examples include PAK6, which is a gene central to signal transduction and cellular regulation. PAK6 is involved in several cellular processes, such as cytoskeletal dynamics, cell motility, gene transcription, and death and survival signaling, and is highly expressed in several brain-tissues (https://gtexportal.org). Another notable example is MPPED1, proposed as the most abundant transcript in the brain [74], particularly in frontal cortex and cerebral cortex, based on GTEx, HPA (https://www.proteinatlas.org), and FANTOM5 [75].

While exploring the tissue-specific gene expression and regulation database (GTEx), we identified additional 53 eQTL SNPs, most of which indicated expression in several tissues of the brain, muscle and nerves (S7 Table). To capture additional functional information, we used FUMA analysis, as described in the methods and the result sections (Fig 6). Two loci were probed for their chromatin effects: rs178850 (p-value of 2.02x10^-8^, in the intronic region of RAB11FIP4 gene, identified by three-way dependency: <*Adaptive*, *lambda*, SNP>); and rs6672510 in the intronic region of PLD5 gene (4.46x10^-8^; identified by two-way dependency; <*lambda*, SNP>) (Fig 6). Both variants are indicated as having intra-chromosome interactions using the chromatin interaction mapping data.

It is interesting that while RAB11FIP4 gene expression is not exclusive to the brain, its expression in brain tissues is higher than in all other tissues reported by GTEx database. RAB11FIP4 has the highest expression in cortex and frontal cortex. Note also that the neighboring gene NF1 (containing the oligodendrocyte myelin glycoprotein gene, OMG) is well known to affect neural growth, and is highly expressed in brain and thyroid. As illustrated in Fig 6A, eQTL mapping of RAB11FIP4 gene identified three genomic regions in chromosome 17, MIR4724, CTD-2349P21.9, and RHBDL3. The non-coding micro RNA MIR4724 is involved in post-transcriptional regulation of gene expression. The non-coding transcript CTD-2349P21.9 is highly expressed in several brain tissues–it has the highest expression in cerebellar hemisphere and cerebellum (responsible for coordination and voluntary movement) compared to all 53 tissue types reported in GTEx database. A similar pattern is observed for RHBDL3 gene. Expression of this gene is the highest in brain tissues, particularly in frontal cortex and cortex (responsible for cognition, memory, and language).

Additionally, four genes, UTP6, CTC-542B22.2, COPRS, RP11-848P1.5, linked *via* chromatin interaction to the RAB11FIP4 locus, were shown to have high expression profiles in all brain tissues (https://gtexportal.org). Taken together all these relationships point strongly to their important role in neurological and growth development in early stages of life.

High expression levels in several brain tissues in GTEx database are also observed for PLD5 gene, with the highest expression in cerebellar hemisphere second to aorta tissues followed by cerebellum. Additionally, chromatin interaction pointed to five genes–AL590483.1, ZBTB18, EXO1, CHML, KMO–with expression levels in several tissues of the brain. Two of these genes, AL590483.1 and ZBTB18, are also expressed highest in cerebellar hemisphere and cerebellum. Expression in cerebellar hemisphere and cerebellum points to the potential role of these genes in movement and activity, fully consistent with our finding of its pleiotropic effect on neurological and growth phenotypes.

While the wide range of information that is integrated here suggests several intriguing conclusions, primarily that the brain-specific, or fetal/placenta-specific character of most of the implicated genes points to brain development as central to growth and infant neurological development, the outstanding weakness of this study is that, to our knowledge, there is no comparable data set that can be used for cross validation. While the number of GUSTO subjects is substantial, it was not statistically sufficient, resulting in some of the candidate relations included in the collection of evidence to be on the border of significance when considered alone. The arguments in favor of collecting a large number of candidate relations, including those that are borderline significant, are substantial if any patterns can be ascertained. This integration of the evidence from our analysis and the knowledge from previous work has allowed us to consolidate such a body of evidence related to neurological development and fetal and early childhood growth in healthy infants that should provide the basis for many future investigations. The results of this study thus represent an initial effort to implement multi-variable genetic analyses to generate a collection of genetic results that can be marshalled to form specific biological hypotheses that need further examination. Further studies will need to provide some validation from independent data sets, as well as capturing existing biological evidence of developmental pathways involving the identified gene candidates and regulatory networks.

## 4. Methods

### 4.1. Data description

#### 4.1.1. Description of key features of the GUSTO data

The GUSTO study of Singapore is the one of the most comprehensive birth and parent-offspring longitudinal cohort studies. It focuses on phenotypic measurements, genetic and epigenetic observations and medical records with detailed study from gestation through the early years of the child’s life [3, 4].

The primary purpose of the GUSTO cohort study is to evaluate the role of developmental factors and influences, including genetic and environmental factors, that affect growth and health. The other objectives are to identify maternal effects on offspring and association with early lifestyles and nutrition that may influence growth and neurocognitive development.

The GUSTO study is an ongoing cohort study that began in 2009. The pregnant women aged 18 years and above were recruited when they attended their first trimester antenatal dating ultrasound scan clinic at Singapore’s two major public maternity units, the National University Hospital (NUH) and the KK Women’s and Children’s Hospital (KKH) between June 2009 and September 2010. The mothers had to be Singapore citizens or permanent residents with Chinese, Malay or Indian ethnicity and homogeneous parental ethnic background, intending to deliver in NUH or KKH and to reside in Singapore for the following 5 years. Mothers receiving chemotherapy, psychotropic drugs or who had type 1 diabetes mellitus were excluded from the study. The women also agreed to donate birth tissues to the study at delivery, *i*.*e*., cord blood, cord, and placenta.

*Ethics approval and consent*. Written informed consent was obtained from all women who participated in the study. Approval for the study was granted by the ethics boards of both KKH and NUH in Singapore. These boards are the Centralized Institute Review Board and the Domain Specific Review Board, respectively.

The recruitment of the mothers for GUSTO cohort study was completed in September 2010. 1,163 pregnant women were recruited: 56% of parents were Chinese, 26% were Malay, and 18% were Indian. The women were on average 30 years old, ranging between 18 and 46 years.

Women recruited in the first trimester returned to the hospital at 19–21, 26–28 and 32–34 weeks of gestation for ultrasound scans to assess gestational age and growth. Detailed interviews were conducted in the clinic at the time of recruitment, and at about 26–28 weeks gestation. Birth tissues were obtained, and anthropometric measurements of the newborn were conducted within 24 hours of birth. During infancy, the babies were examined at home at 3 weeks, 3 months, and every 3 months thereafter until 15 months of age. The children were then evaluated at the clinic at 18, 24, and 36 months, and the Bayley scale scores used in this work were acquired at 24 months.

#### 4.1.2. SNPs, Phenotype data, and growth parameter data used for the analysis–results elucidated in the GUSTO study

The acquired genotype data consists of 933,886 SNPs from 1,073 infants and parents, as previously reported. The phenotype data consists of 10,378 features from 1,237 infants and parents, which include ethnicity, gender, anthropometric measurements, socioeconomic measurements, and neurological phenotypes such as Bayley scales of infant and toddler development, Brief Infant Sleep Questionnaire (BISQ), Child Behavior Check List (CBCL), and Infant Toddler Social Emotional Assessment (ITSEA). The phenotypes data, of particular note the Bayley scale data, were collected by professionals.

In this paper, we only focused on the infant data. Moreover, in this paper we only consider three types of information: genotype (SNPs), neurological, and growth data. The neurological data consists of the following subsets:

1. Bayley, 60 features: 5 categorical and 55 numerical,
2. BISQ, 13 features: 4 categorical and 9 numerical,
3. CBCL, 341 features: 296 categorical and 45 numerical,
4. ITSEA, 153 features: 152 categorical and 1 numerical,

where categorical features are qualitative variables that take on non-numerical values (words). All neurological data used in this paper was measured from 6 months to 48 months. Furthermore, when analyzing the genetic component of the infant development, we used only 5 aggregate or composite Bayley scales: Cognitive, Language, Motor, Adaptive Behavior, and Social-emotional, measured at 24 months. We simply refer here to Bayley phenotypes when directly referring to these 5 aggregate scales.

The growth data we used consists of three parameters of Gompertz-like growth model fits that describe fetal head circumference growth as a function of gestational age. The growth parameters were available for 1,053 infants (see Section 4.2.3).

We analyzed multiple pairwise and three-variable dependencies. For each type of dependency, we only used infants with values in all analyzed variables, and vice versa, we removed genetic variables (SNPs) with missing values (see Section 4.2.4). The following is the summary of the different types of dependencies we analyzed and the corresponding number of SNPs and samples used in each type:

1. Pairwise dependencies of the type <Neuro, Growth>, 281 infants
2. Three-variable dependencies of the type <Neuro, Growth, Growth> and <Neuro, Neuro, Growth>, 281 infants
3. Pairwise dependencies of the type <Bayley, SNP>, 495,719 SNPs, 433 infants
4. Pairwise dependencies of the type <Growth, SNP>, 448,658 SNPs, 1053 infants
5. Three-variable dependencies of the type <Growth, Bayley, SNP>, 495,719 SNPs, 428 infants

### 4.2. Preprocessing of data

**Fig 10:**
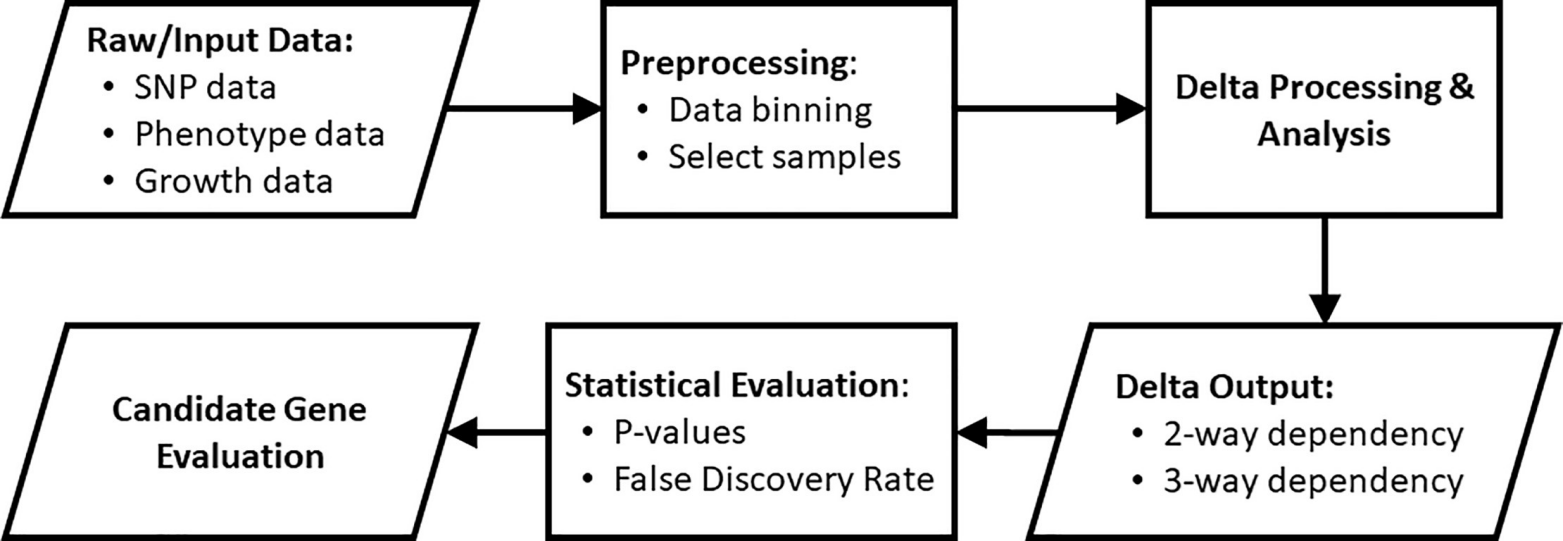
Flow chart of the process of selecting gene candidates using our dependency measures. The measures of multi-variable dependencies and Delta are described in Section 4.1.3, and the preprocessing of phenotypes and SNP data are described in Section 4.2. The statistical evaluation is explained in Section 4.3.

The flow of the data analysis using Delta measures is shown schematically in Fig 10. There are three principal stages of the analysis: Preprocessing, Delta Computation, and Statistical Evaluation, leading to genetic candidates. Note that by “gene candidates” we refer to nearby genes to the implicated SNPs. Although we use the closest gene to a SNP to indicate a locus, in each case we also examine the region of the genome to determine if there are other nearby genes of interest.

In the preprocessing stage, we generate input data files for the Delta software from the raw data of SNPs, neurological phenotypes, and growth parameters. The input data must be discrete, represented by positive integers, so all continuous data must be binned. To account for specific structures and properties of data subsets, SNPs, neurological phenotypes, and growth parameters were preprocessed individually.

#### 4.2.1. Preprocessing SNP data

**Fig 11:**
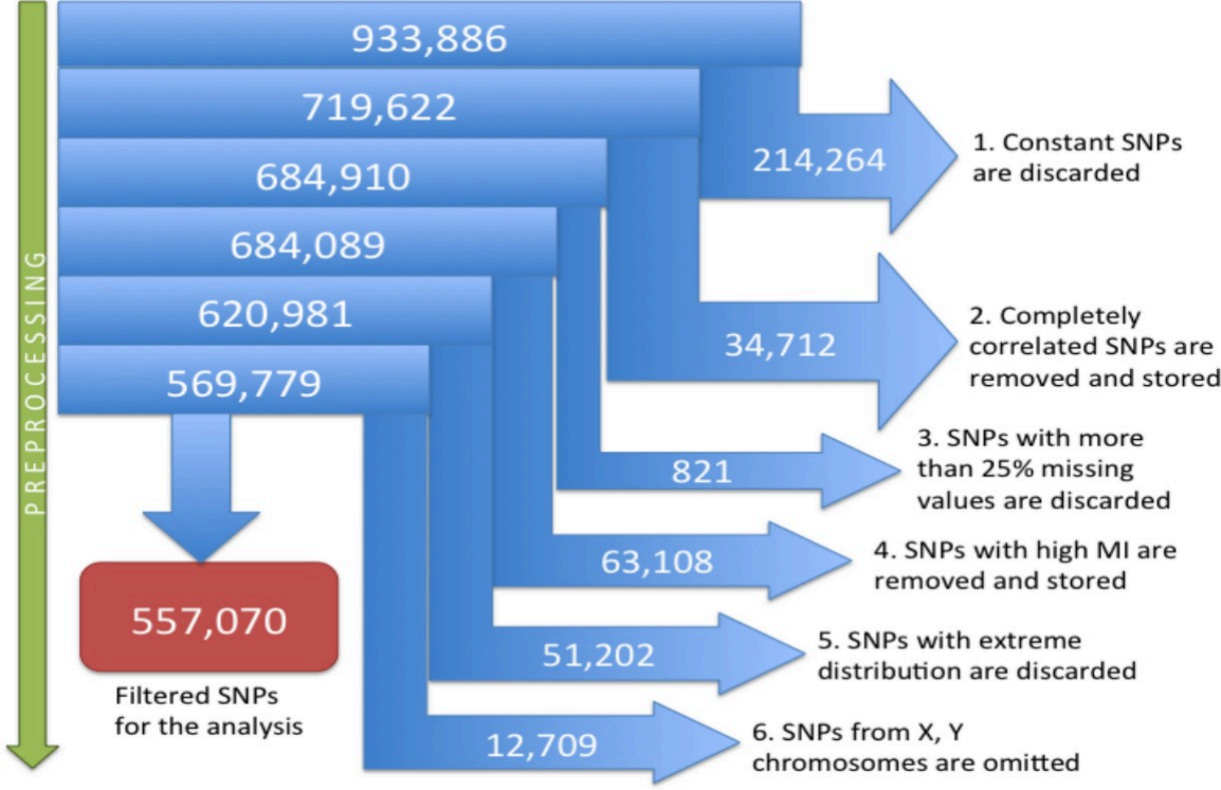
The preprocessing steps of the genotype data showing the number of SNPs removed from Delta analysis.

The genotype data for the infants of the GUSTO project consists of 933,886 SNPs, obtained using the Illumina Omniexpress & exome array. SNPs with call rates < 95%, or minor allele frequency < 5%, or those that failed Hardy–Weinberg Equilibrium test were excluded from the analysis. Out of 1071 infant subjects, 2 infants with no genotype information were removed. To prepare the data for our analysis, we reduced the amount of redundancy and gaps in the SNP data. Fig 11 summarizes the SNP filtering steps.

Preprocessing of genotype data starts with the removal of constant SNPs that show no variation among all infants in the study (Step 1). At Step 2, the completely correlated SNPs, *i*.*e*., SNPs that are in complete linkage disequilibrium, are “collapsed” for the dependency analysis, keeping one SNP per correlated group of SNPs. Note that these correlated SNPs are omitted only at the stage of dependency detection, since they do not add any more information about dependency and put back into the analysis once the candidate dependencies have been detected. Preprocessing continues with Step 3 by removing SNPs with more than 25% of missing values. At Step 4, we compute mutual information for all pairs of SNPs and “collapse” SNPs with high (over 1.2) mutual information, keeping one representative of each mutual information cluster. At Step 5, we remove SNPs with extreme distribution of genotypes, which are the SNPs that show no variation in more than 95% of infants. In our analysis, because of the gender differences in the SNP variables and the potential differences between male and female growth rates, we decided to eliminate effects of gender; that is, we looked only for those effects that were common, and therefore Step 6 of the preprocessing removes the SNPs from *X*, *Y* chromosomes. Fig 11 shows the number of removed or collapsed SNPs at each step after all the preprocessing steps. There are 557,070 SNPs in 1071 infants selected for the dependency analysis.

**Fig 12:**
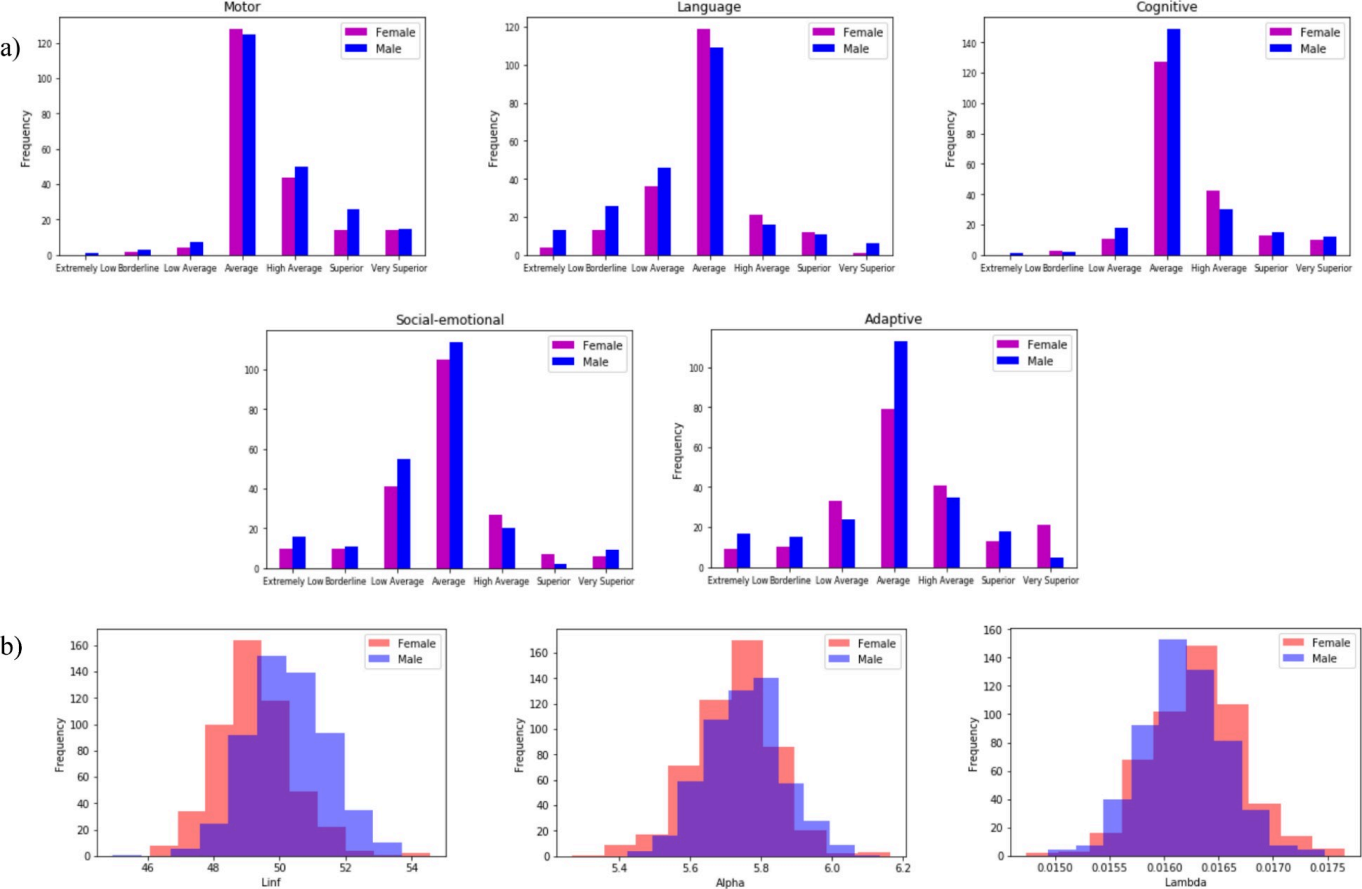
Distribution of SNPs in the X and Y region for Bayley’s and growth phenotypes. **a**) The distributions of Bayley phenotypes by male and female infants used for the two-variable <Bayley, SNPs> analysis. **b**) The distributions of Growth phenotypes by male and female infants used for the two-variable <Growth, SNPs> analysis.

We performed the dependency analysis separately for SNPs sets from *X*, *Y* chromosomes, including the pseudoautosomal regions, which were removed during preprocessing at Step 6 for male and female infants. The number of male and female subject as well as the number of SNPs used in each two-way and three-way dependency analysis are provided in S5 Table. Fig 12A and 12B shows the distributions of growth and Bayley phenotypes divided by male and female infants used in the dependency analysis. The differences in the distributions between males and females were observed in growth phenotypes *linf* and *lambda*. However, no significant differences between male and female distributions were detected in either Bayley scale or the *alpha* growth phenotypes.

#### 4.2.2. Preprocessing neurological data

The original phenotype data contains 10,378 features, consisting of categorical (taking on a small set of word answers to questionnaires) and numerical phenotypes. The phenotype data includes various observations such as anthropometric measurements and questions about child environment at different time points for 1,237 individuals. We focused on neurodevelopmental data of each child, consisting of Bayley scales, BISQ, CBCL, and ITSEA. Preprocessing of categorical and numerical neurological phenotypes was done separately. In this study we only used the Bayley scale data.

During preprocessing, we removed categorical phenotypes with more than 10 different categories. Having variables with too many possible values strongly weakens the statistical power of dependency detection. To convert the categorical phenotypes into the input for the dependency analysis, their values were encoded using integers.

To preprocess the numerical phenotypes, we examined the distributions of values and it was commonly appropriate to discretize their values into four bins as follows: (-∞,*l*], (*l*,*μ*], (*μ*,*r*], (*r*,∞), where *μ* is the mean and *l* and *r* are the medians of the values below and above of the mean. Each bin was encoded with an integer. Similar to Step 5 of the SNP preprocessing (Fig 11), categorical and numerical phenotypes that have the same value in more than 95% of infants were removed.

#### 4.2.3. Preprocessing growth data and the growth model description

A non-linear mixed effects (NLME) Gompertz-like model was used to fit the growth data (head circumference) to obtain three parameters characterizing growth of each individual subject. These describe the final growth limit for head circumference (*linf*), nonlinearity of head growth deceleration at around 20 weeks gestation (*lambda*), and an early velocity of head circumference (*alpha*). The growth of each infant is represented by these three parameters. We further discretize these growth parameters using the same approach as in the case of numerical neurological phenotypes.

*Model description*. Subject head circumference growth trajectories from early pregnancy to early childhood (ended at 54 months) were characterized using the following NLME model

$$Y_{ij}=[L_{p}+L_{i}]e^{-[\alpha_{p}+\alpha_{i}]\exp\left[-\beta_{p}t_{ij}(1-\theta_{p}[1-e^{-[\lambda_{p}+\lambda_{i}]t_{ij}}])\right]}+\varepsilon_{ij}.$$

This functional form was selected from among several candidate models based on the AIC and examination of residual plots. As in the standard three-parameter Gompertz model [75], fixed-effect (population-level) parameters are used to characterize the growth limit (*L*_*p*_), ratio of lower to upper limit (*α*_*p*_), and growth rate (*β*_*p*_) for the entire population. To this model, two more fixed-effect parameters are added to characterize the rate (*θ*_*p*_) and nonlinearity (*λ*_*p*_) of the deceleration in growth rate that begins around 20 weeks gestation. Finally, to account for variation in growth trajectories between subjects, subject-level effects were inferred for each subject for these parameters: growth limit (*L*_*i*_), ratio of lower to upper limit (*α*_*i*_) and nonlinearity of growth deceleration (*λ*_*i*_), (*linf*, *alpha*, *lambda*). The subject level random effects were assumed to be uncorrelated with each other or with the error term (*ε*_*ij*_).

*Parameter Estimation for growth model*. The model was fit to head circumference data measured on subjects from four birth cohort studies included in the Bill and Melinda Gates Foundation knowledge integration database. The combined dataset included a total of *N* = 11,818 subjects, each contributing between 2 and 18 measurements of head circumference, measured between 8 and 290 weeks post-menstrual age [77]. Note that a 5-parameter growth curve was fit to the entire population and allowed for subject-specific deviations from the population mean curve in 3 parameters (*linf*, *alpha*, *lambda*) as shown in Table 10. After that the individual estimates for these 3 parameters across 1,191 subjects of GUSTO cohort study were used as the input growth data in our downstream dependency analysis. Calculations and fitting to the individual infant data were completed using the *nlme* package [78] in the R statistical software (https://CRAN.R-project.org/package=nlme [79]).

**Table 10:** Parameter estimates for Gompertz model with nonlinear deceleration fit to head circumference data from children between early pregnancy and earlys childhood.

a) **Fixed Effect (population level) Parameter Estimates**					
	***Estimate***	***Std Error***	***df***	***t-value***	***p-value***
*linf*	49.96582	0.03487290	62416	1432.780	0
*alpha*	5.62027	0.01535200	62416	366.0939	0
*beta*	0.10450	0.00025640	62416	407.5520	0
*theta*	0.75950	0.00148836	62416	510.2935	0
*lambda*	0.01667	0.00007920	62416	196.5385	0
b) **Subject Effect Parameter Estimates**					
	***Var*. *Comp*.**				
*linf*	1.305845				
*alpha*	0.1648373				
*lambda*	0.0005637008				
*residual*	0.7218403				

a) Fixed parameter estimates. The top row shows maximum likelihood estimate for the growth limit parameter *linf*, the estimated standard error for the maximum likelihood estimator, and the corresponding degrees of freedom (*df*). *t-value* is the test statistic (in this case a *t* statistic) for the null hypothesis that *linf* = 0. Because the degrees of freedom are so large, the null distribution of the test statistic is essentially a normal distribution, and p-value = 1−2*ϕ*(|t*|), where *ϕ* is the standard normal cdf. The additional rows follow the same pattern for the other fixed effect (i.e., population-level) parameters. b) Subject parameter estimates. The maximum likelihood estimate of the variance parameter of the distribution of the subject-level random effects of the growth limit parameter is shown in the first row. The additional rows represent estimates for the other subject-level variance components as labelled.

#### 4.2.4. Missing data: Selecting optimal subsets by linear programming

Once the data preprocessing is complete, we needed to face the problem of missing data among the variables. When performing the dependency detection using Delta software, missing data can cause significant fluctuations and decrease reliability of the results. We therefore selected subsets of data that reduce the number of missing values while keeping as many subjects and variables as possible. Many clustering and bi-clustering methods are suitable for this task, but we decided to use a simple linear programming optimization method.

Linear programming is a method for optimization of a linear objective function *z*, subject to linear inequality constraints allowing us to maximize the number of variables and subjects while minimizing the amount of missing values (assuming all missing values are encoded as -2) as follows:

maximizez=n+m. (1)

$$subject\;to\;\sum_{j=1}^{m}b_{ij}>-2m,\;(i=1,\ldots ,n),$$ (2)

$$and\;M_{j}/n\leq \tau ,(j=1,\ldots ,m),$$ (3)

where *n* is the number of subjects, *m* is the number of variables, *b*_*ij*_ is a binned value of a variable *j* in a subject *i*, *M*_*j*_ is the number of missing data in a variable *j*, and *τ* is a threshold for missing values for each variable.

Constraint (2) ensures that we remove a subject if each of the *m* variables has missing values. Note that constraint (2) assumes that a missing value is represented by -2 and all other values are non-negative, therefore, if a subject has at least one variable with non-missing (non-negative) value (out of *m* variables), then the sum of binned values of all variables would be higher than -2*m*. However, if values of all *m* variables are missing for a subject, the sum of binned values is equal to -2*m*.

Constraint (3) ensures the amount of missing data (the fraction *M*_*j*_/*n*) is below a certain level (*τ*) for each variable. In order to find the solution to our linear programming problem, we need to provide the value of *τ*. During the analysis of the pairwise dependencies between the neurological phenotypes and the growth parameters, we used *τ* = 25%. For the analysis of all other types of dependencies *τ* was set to 0.

Using our linear optimization method, we selected optimal subsets of variables and samples to be used in the dependency analysis, as described in Section 4.3.

### 4.3. Multi-variable dependency using information theory methods

Biological data is filled with various dependencies since it is obtained from complex systems with many interactions. Therefore, we need detection of multivariable dependencies of diverse kinds in order to effectively analyze biological data. We have recently introduced an information theory-based set of dependency measures and implemented the discovery of multivariable dependencies in a large set of variables capitalizing on a distinct advantage of separating the detection of the dependence from defining the nature of the dependence [13–15]. In general, information theory measures have several advantages: they are inherently model-free and non-parametric in nature, and they exhibit only modest sensitivity to undersampling [80]. We have described these methods in several papers previously [13–15] and will briefly summarize the methods here for up to three variables, which is the maximum number used in this paper.

Our information theory-based method iteratively searches through a set of variables (e.g., SNPs, growth parameters) and systematically detects strong dependencies with increasing degree, starting with the pairwise dependencies, then three-variable dependencies, and so on. In this paper, we limited our method to only pairwise and three-variable dependencies. To measure a general dependence between two variables, *X* and *Y*, we use mutual information *I*(*X*,*Y*), defined as

I(X,Y)=H(X)+H(Y)−H(X,Y), (4)

where *H*(*X*) and *H*(*Y*) are single entropies of variables *X* and *Y* and *H*(*X*,*Y*) is their joint entropy.

To measure a general dependence between three variables, *X*, *Y*, and *Z*, we use symmetric delta $\bar{\Delta }(X,Y,Z)$. Before providing the definition for the symmetric delta, we need to introduce interaction information, which was proposed long ago as a multivariable generalization of mutual information [80]. For three variables interaction information *I*(*X*,*Y*,*Z*) is defined as

I(X,Y,Z)=I(X,Y)−I(X,Y|Z). (5)

Given Eq 5, we define differential interaction information Δ as the difference between values of successive interaction information arising from adding variables:

$$\begin{array}{c} \Delta_{X}=I\left(X,Y,Z\right)-I\left(Y,Z\right)\\ \Delta_{Y}=I\left(X,Y,Z\right)-I\left(X,Z\right)\\ \Delta_{Z}=I\left(X,Y,Z\right)-I\left(X,Y\right) \end{array}$$ (6)

Here Δ_*X*_ is called the asymmetric delta for the target variables *X*. In order to detect a fully cooperative dependence among the variable set, we want any single measure to be symmetric. As a result, we define a more general measure $\bar{\Delta }$, called the symmetric delta (or delta), by multiplying Δ with all possible choices of the target variable:

$$\bar{\Delta }(X,Y,Z)=\Delta_{X}\cdot \Delta_{Y}\cdot \Delta_{Z}.$$ (7)

The key property of the symmetric delta is that if any of the three variables is independent of the others, then the measure is zero. Note that although we focus on three-variable case here, this definition can be generalized to any number of variables.

Note also that the asymmetric deltas are related in a subtle way:

$$\begin{array}{c} \Delta_{X}+I\left(X,Y\right)+I\left(X,Z\right)=\Omega \\ \Delta_{Y}+I\left(X,Y\right)+I\left(Y,Z\right)=\Omega \\ \Delta_{Z}+I\left(X,Z\right)+I\left(Y,Z\right)=\Omega \end{array}$$ (8)

where Ω is the multi-information, called total correlation when introduced by Watanabe [81]. This measure captures the full dependence, for all variable subsets, and is zero only if all the variables are independent. For three variables the multi-information is defined as

$$\Omega =\sum_{v\in \left\{X,Y,Z\right\}}H\left(v\right)-H\left(X,Y,Z\right).$$ (9)

High absolute values of the Delta measure or mutual information indicate that the corresponding variables are collectively interdependent. On the other hand, small values of Delta and mutual information indicate that all variables are approximately independent. Note that both measures are symmetric under permutation of variables. By our definition, values of three-variable Delta are negative, while values of mutual information are always positive. Mutual information and Delta can detect not only linear correlations, but any nonlinear relationships among variables.

The dependency can sometimes be usefully interpreted as a relation among variables described as logical functions such as AND, OR and XOR, albeit for more than a binary alphabet. The Delta can effectively detect an XOR type of function, for example, which has no pairwise dependence and therefore no mutual information between any pairs of its variables.

The Delta measures and the methods for optimally computing these measures across large data sets have been implemented in software we refer to here as the “Delta software”.

### 4.4. Statistical significance of dependency results

#### 4.4.1. Delta p-values by permutation test

In order to estimate the significance of the dependency scores calculated by Delta software, we carried out a permutation test by generating randomly shuffled input files and examining the distributions of resulting scores. We used two criteria for analyzing the statistical significance: (1) its information score’s p-value, and (2) whether the score is above or below the threshold calculated from the maximal random scores. The information scores obtained as described below were both those not adjusted for ethnic confounding and the adjusted values (see section 4.4.2)

To obtain unadjusted p-values of dependency scores we follow the permutation strategy proposed by Churchill and Doerge [82]: we shuffle the input data, breaking the connections between variables, compute the dependency scores of all shuffled tuples, and count how many randomized scores are above the original score of interest. We repeat this procedure 1000 times tallying the number of scores above the score of interest. The p-value is then the fraction the exceeding randomized scores take in the total number of tuples times 1000. Note that when determining the statistical significance of pairwise dependencies, we permuted the values of the phenotype variables thus breaking the phenotype-SNP relationships in the data, while at the same time preserving all linkages between SNPs. Similarly, for three-variable dependencies, we independently permuted the values of Growth phenotypes and Bayley phenotypes, thus breaking the relationships not only between phenotype and genotype variables, but also between Growth and Bayley phenotypes. Note that these p-values are unadjusted, not accounting for multiple hypothesis testing.

Although we decided not to perform conventional multiple hypothesis testing, since the goal of our paper is not to search for causal SNPs, we acknowledge that due to the large number of SNPs in our analysis the number of false positive may be high. To shed some light on the amount of false-positives in our analysis, we followed the approach of Churchill and Doerge [82] and calculated Family-Wise Error Rate (FWER). To calculate FWER, we find an absolute maximum randomized score for each shuffle, construct a distribution of 1000 maximum randomized scores (since we performed 1000 shuffles), and find how many scores from this distribution are above the original score of interest.

FWER is the probability of getting *at least one* false positive result given a large number of comparisons we made (the number of SNPs in the analysis), so it is highly conservative and it is not a surprise that many of our top dependencies have a high value of FWER. Nevertheless, a number of our results showed low values of FWER. Furthermore, we used FWER to compare the confounding effect of ethnicity on our pairwise dependencies (see the next section).

#### 4.4.2. Ethnicity of subjects and possible confounding effects

Since the subjects selected for data analysis are of three ethnicities, and it is clear that there are allele frequency differences among the Chinse, Indian and Malay populations, it was necessary to examine the possibility of confounding effects of the ethnic differences on our genetic results. Because the number of subjects is rather small, we divided the subject population into two groups: (1) Chinese, and (2) Indian + Malay. The full population of infants with no missing Bayley phenotypes had 433 subjects, 258 of which were Chinese and 175 were Indian or Malay. We then used a binary variable ε_i_ indicating ethnicity (Chinese or Indian-Malay) to examine the relevant conditional probabilities, and determined the confounding effects of the ethnicity on the mutual information measures. The probabilities sum to one: P(ε_1_) + P(ε_2_) = 1,

For the set of SNPs, *S*, and the set of Bayley phenotypes, *B*, and

$$\pi (b|s)=\sum_{i}p(b|s,\varepsilon_{i})$$

The adjusted mutual information then is

$$\tilde{I}(b,s)=\sum_{s\in S,b\in B}p(s)\pi (b|s)log(\pi (b|s))-\sum_{s\in S,b\in B}p(s)\pi (b|s)log(p(b))$$

Table 11 shows the values of adjusted mutual information for the dependencies from Tables 2 and 3.

**Table 11:** Results for dependencies adjusted for the confounding effect of ethnicity. A. is for the Bayley phenotypes, and B is for the growth phenotypes.

**A**									
**Bayley Phenotype**	**SNP**	**Gene**	**MI**	**P-value (MI)**	**FWER (MI)**	**Adjusted MI**	**P-value (Adj.)**	**p-value change (%)**	**FWER (Adj.)**
Motor	rs10833478	NELL1	0.0843	3.41E-07	0.142	0.0846	3.25E-07	4.7%	0.135
Motor	rs645026	YEATS4	0.0832	4.40E-07	0.180	0.0828	4.78E-07	8.7%	0.197
Adaptive	rs7387693	MTMR7	0.0847	9.54E-07	0.359	0.0844	1.05E-06	9.9%	0.384
Adaptive	rs7462219	MTMR7	0.0807	2.67E-06	0.693	0.0802	2.96E-06	11.1%	0.735
Adaptive	rs4955988	CACNA2D3	0.0817	2.13E-06	0.617	0.0812	2.38E-06	11.5%	0.651
Adaptive	rs1291359	HTR7P1	0.0829	1.55E-06	0.506	0.0839	1.17E-06	24.2%	0.414
Language	rs11658800	ELAC2	0.0803	2.48E-06	0.670	0.0828	1.31E-06	46.9%	0.437
Language	rs7239403	SMIM21	0.0803	2.51E-06	0.675	0.0853	7.42E-07	70.5%	0.288
Social-Emotional	rs12434723	C14orf177	0.0805	2.61E-06	0.680	0.0858	7.26E-07	72.2%	0.272
Social-Emotional	rs11628108	C14orf177	0.0826	1.56E-06	0.510	0.0881	3.59E-07	77.0%	0.143
Motor	rs7155811	TMEM260	0.0772	2.16E-06	0.610	0.0940	5.45E-08	97.5%	0.020
Social-Emotional	rs1161106	LOC100507175	0.0811	2.28E-06	0.634	0.0780	4.78E-06	109.2%	0.876
Motor	rs1449848	CPNE8	0.0772	2.16E-06	0.607	0.0645	5.19E-05	2305.1%	1.000
B									
**Growth Phenotype**	**SNP**	**Gene**	**MI**	**P-value (MI)**	**FWER (MI)**	**Adjusted MI**	**P-value (Adj. MI)**	**p-value change (%)**	**FWER (Adj.)**
Linf	rs12734338	PPP1R12B	0.1130	2.23E-09	0	0.1141	2.23E-09	0.0%	0
Linf	rs7981995	DACH1	0.0232	7.18E-06	0.936	0.0231	7.57E-06	5.5%	0.944
Alpha	rs7101173	MIR6072	0.0248	2.54E-06	0.619	0.0247	2.71E-06	6.7%	0.643
Linf	rs373680	FBXO33	0.0232	6.94E-06	0.93	0.0235	5.83E-06	16.0%	0.895
Lambda	rs4793500	CASC17	0.0259	1.16E-06	0.358	0.0256	1.46E-06	25.5%	0.427
Lambda	rs564266	NTM	0.0241	4.05E-06	0.771	0.0249	2.47E-06	39.0%	0.620
Lambda	rs773024	OSTF1	0.0236	5.53E-06	0.855	0.0229	8.57E-06	55.0%	0.952
Linf	rs6884117	C5orf22	0.0241	3.86E-06	0.761	0.0234	6.18E-06	60.1%	0.908
Lambda	rs154444	ZNF608	0.0232	7.08E-06	0.916	0.0257	1.33E-06	81.2%	0.404
Linf	rs7071157	PFKFB3	0.0276	4.08E-07	0.157	0.0300	7.13E-08	82.5%	0.031
Linf	rs6570627	UTRN	0.0250	2.20E-06	0.576	0.0240	4.19E-06	90.1%	0.789
Alpha	rs10196354	ERBB4	0.0232	7.23E-06	0.937	0.0284	2.14E-07	97.0%	0.085
Lambda	rs6672510	PDL5	0.0316	3.12E-08	0.013	0.0298	9.81E-08	214.3%	0.038
Alpha	rs9691259	IGFBP3	0.0258	1.30E-06	0.4	0.0232	7.40E-06	469.8%	0.937
Linf	rs6710428	CERS6	0.0264	8.83E-07	0.301	0.0224	1.19E-05	1253.3%	0.993
Lambda	rs7075547	LRRTM3	0.0237	5.15E-06	0.836	0.0043	4.07E-01	7893823%	1

We show the population wide mutual informations and corresponding p-values, with the adjusted mutual informations and p-values, followed by the FWER value. The three sets of SNP-phenotypes separated by thick lines are those for which: upper–the absolute change in p-values is less than 25% of the original; middle–the change in p-value is less than 100%; lower the change in p-value is greater than 100%. Note that there are some dependencies for which the p-values improve on adjustment for ethnicity confounding.

The changes for most of the p-values are rather small, some almost non-existent. However, we note that there are several that improve modestly upon adjustment, and a couple, CERS6 and LRRTM3 for which the confounding effect is dramatic, and significantly reduces this dependency.

### 4.5. Functional genomic analysis

#### 4.5.1. Variant annotation

Functional annotation was carried out using Ensemble Variant effect predictor (VEP; https://www.ensembl.org/vep) for two-way and three-way dependency sets. VEP determines the effect of genomic variants on genes, their transcripts, and protein sequence, as well as regulatory regions. Additional identifiers for each variant generated by VEP includes information such as gene symbol, variant specificity (such as exonic, intronic, UTRs), splice site (donor/acceptor), transcription factor binding sites, synonymous codon changes and frameshift variants.

#### 4.5.2. Regulatory analysis

The majority of the identified variants in the two-way and three-way dependency sets are located in the non-coding regions of the genome including intronic, intergenic, upstream or downstream from genes and in 3’ and 5’ UTRs. We examined their potential effect on regulatory functions using RegulomeDB [31] (http://www.regulomedb.org). RegulomeDB, an integrated database, includes all available ENCODE transcription factor ChIP-seq, histone ChIP-seq, FAIRE, and DNase I hypersensitive site data [83], transcription factor ChIP-seq data available from the NCBI Sequence Read Archive [84–92] as well as a large collection of eQTL [86, 93–100], dsQTL [101], and ChIP-exo [102] data. We queried RegulomeDB using the dbSNP identifiers of our candidate variants, resulting in a set of known, validated and/or predicted regulatory elements. These were categorized based on their potential functional impact and integrated to assemble a network based on common transcription relationships.

#### 4.5.3. Genotype-Tissue Expression identification

Since the phenotypes of interest in our study were neurological and growth related, we examined our set of prioritized gene candidates using the Genotype-Tissue Expression (GTEx v7; http://gtexportal.org) portal, a catalogue of tissue-specific and shared regulatory expression quantitative trait loci (eQTL) variants. This allowed us to acquire additional information on healthy human gene expression patterns in multiple tissues [103]. The output includes the levels of gene expression across all tissue types as well as within tissues, some of which are of interest as they are involved in neurological and growth processes such as expression levels in skeletal muscles, tibial nerve and multiple tissues of brain. GTEx database (http://gtexportal.org) captures the estimated effect size of an eQTL allele on gene expression, which allows for identifying genes, whose expression is affected by genetic variation, providing information on variant’s potential involvement in phenotype.

#### 4.5.4. Regulatory interaction network analysis

The NetworkAnalyzer package [104–106] available in Cytoscape v.3.3.0 (http://www.cytoscape.org) was used for clustering and visualization of both regulatory and direct protein-protein interaction network. No notable results from this are reported. NetworkAnalyzer allows for computing a set of graph parameters for undirected and directed networks. In particular, we used betweenness centrality for clustering and visualization of both regulatory and direct protein-protein interaction networks.

#### 4.5.5. Functional mapping and annotation of genome-wide association studies (FUMA)

To further evaluate the candidate set of variants identified by our two-way and three-way dependency analysis, we used FUMA v1.3.3c [35] (http://fuma.ctglab.nl), which uses a set of statistically significant SNPs as an input and provides functional annotations. FUMA uses data from positional mapping, including eQTL mapping, and 3D chromatin interaction mapping (Hi-C for 14 human tissues including prefrontal cortex and hippocampus), to predict potential regulatory effects from chromatin states at the position of the SNP of interest, and MAGMA gene expression analysis [35], selecting 53 tissue types from GTEx [34]. We used default parameters during the analysis. Out of the set of resulting SNPs, we focused on two interesting loci located in *RAB11FIP4* and *PLD5* (see the results section and Fig 6).

## Supporting information

S1 Table The top 77 significant SNPs associated with growth model parameters (*linf*, *lamda* and *alpha*) and Bayley neurological phenotypes using three-variable dependency by asymmetric Delta, ∆_1. The target variable in this table is a growth phenotype.
(XLSX)

S2 Table The top 106 significant SNPs associated with growth model parameters (*linf*, *lamda* and *alpha*) and Bayley neurological phenotypes using three-variable dependency by asymmetric Delta, ∆_2. The target variable in this table is a neurological phenotype.
(XLSX)

S3 Table The Top 117 significant SNPs associated with growth model parameters (*linf*, *lamda* and *alpha*) and Bayley neurological phenotypes using three-variable dependency by asymmetric Delta, ∆_3. The target variable in this table is the SNP.
(XLSX)

S4 Table The top 10 significant SNPs specific to X and Y region associated with each phenotype(s): Growth model parameters (*linf*, *lamda* and *alpha*) and Bayley neurological phenotypes for both pair-wise and three-variable dependency. SNPs that occur in more than one category (gender or phenotype) are identified with the same color. The "exm" SNPs that were excluded from the downstream analysis are highlighted in red.
(XLSX)

S5 Table Preprocessing of SNPs in X and Y chromosomes. (XLSX)

S6 Table The RegulomeDB score for 148 candidates associated with either neurological, growth parameters or both using Mutual Information and Delta3 dependency measures respectively. Variants that returned "no data" are not included.
(XLSX)

S7 Table Tissue specific gene expression evidences using GTEx and regulatory function scores from RegulomeDB for the candidate SNPs. (XLSX)

S8 Table Selected SNPs in the top 3 scoring categories of RegulomeDB, with the most likely regulatory functions. (XLSX)

S1 Fig The one-SNP three-gene structure. The SNP rs178850 has the best p-value (2x10^-8^) of those identified in a three-way dependency with Adaptive (Bayley phenotype) and *lambda* (growth phenotype). This SNP is located in the intronic region of RAB11FIP4 gene on chromosome 17 and is also very close to NF1 and OMG, which is located within NF1. All three of these genes affected by rs178850 are highly expressed in brain.
(PDF)
