# Supplementary figures and images for: Complex genetic dependencies among growth and neurological phenotypes in healthy children: Towards deciphering developmental mechanisms
Source: PLoS One. 2020 Dec 3;15(12):e0242684. doi: 10.1371/journal.pone.0242684 (PMC7714163; doi:10.1371/journal.pone.0242684)

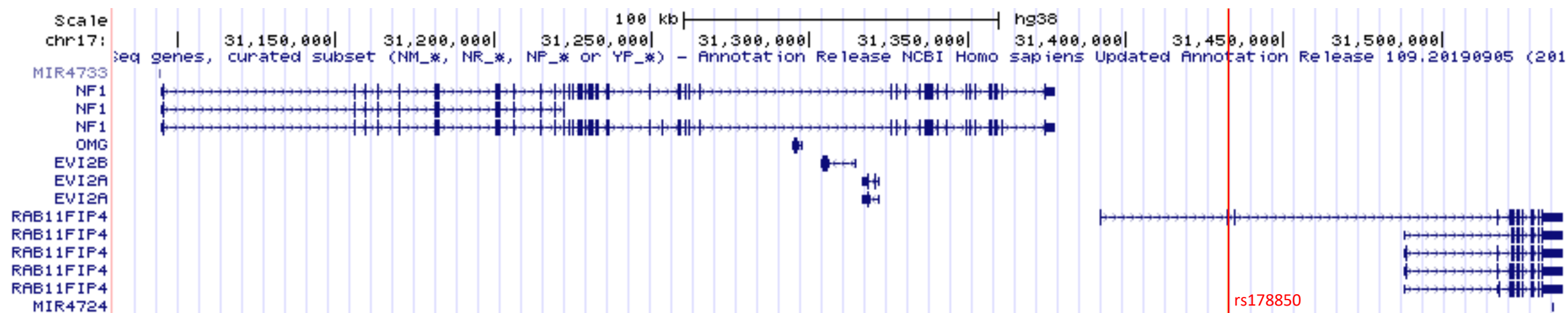

Supplement: S1 Fig — The SNP rs178850 has the best p-value (2x10^-8^) of those identified in a three-way dependency with Adaptive (Bayley phenotype) and lambda (growth phenotype). This SNP is located in the intronic region of RAB11FIP4 gene on chromosome 17 and is also very close to NF1 and OMG, which is located within NF1. All three of these genes affected by rs178850 are highly expressed in brain. (PDF) [file pone.0242684.s009.pdf]
